# Epigenetic reprogramming of a distal developmental enhancer cluster drives *SOX2* overexpression in breast and lung adenocarcinoma

**DOI:** 10.1093/nar/gkad734

**Published:** 2023-09-22

**Authors:** Luis E Abatti, Patricia Lado-Fernández, Linh Huynh, Manuel Collado, Michael M Hoffman, Jennifer A Mitchell

**Affiliations:** Department of Cell and Systems Biology, University of Toronto, Toronto, Ontario, Canada; Laboratory of Cell Senescence, Cancer and Aging, Health Research Institute of Santiago de Compostela (IDIS), Xerencia de Xestión Integrada de Santiago (XXIS/SERGAS), Santiago de Compostela, Spain; Department of Physiology and Center for Research in Molecular Medicine and Chronic Diseases (CiMUS), Universidade de Santiago de Compostela, Santiago de Compostela, Spain; Princess Margaret Cancer Centre, University Health Network, Toronto, Ontario, Canada; Laboratory of Cell Senescence, Cancer and Aging, Health Research Institute of Santiago de Compostela (IDIS), Xerencia de Xestión Integrada de Santiago (XXIS/SERGAS), Santiago de Compostela, Spain; Princess Margaret Cancer Centre, University Health Network, Toronto, Ontario, Canada; Department of Medical Biophysics, University of Toronto, Toronto, Ontario, Canada; Department of Computer Science, University of Toronto, Toronto, Ontario, Canada; Vector Institute for Artificial Intelligence, Toronto, Ontario, Canada; Department of Cell and Systems Biology, University of Toronto, Toronto, Ontario, Canada; Laboratory Medicine and Pathobiology, University of Toronto, Toronto, Ontario, Canada

## Abstract

Enhancer reprogramming has been proposed as a key source of transcriptional dysregulation during tumorigenesis, but the molecular mechanisms underlying this process remain unclear. Here, we identify an enhancer cluster required for normal development that is aberrantly activated in breast and lung adenocarcinoma. Deletion of the SRR124–134 cluster disrupts expression of the *SOX2* oncogene, dysregulates genome-wide transcription and chromatin accessibility and reduces the ability of cancer cells to form colonies *in vitro*. Analysis of primary tumors reveals a correlation between chromatin accessibility at this cluster and *SOX2* overexpression in breast and lung cancer patients. We demonstrate that FOXA1 is an activator and NFIB is a repressor of SRR124–134 activity and *SOX2* transcription in cancer cells, revealing a co-opting of the regulatory mechanisms involved in early development. Notably, we show that the conserved SRR124 and SRR134 regions are essential during mouse development, where homozygous deletion results in the lethal failure of esophageal–tracheal separation. These findings provide insights into how developmental enhancers can be reprogrammed during tumorigenesis and underscore the importance of understanding enhancer dynamics during development and disease.

## Graphical Abstract

**Graphical Abstract:**
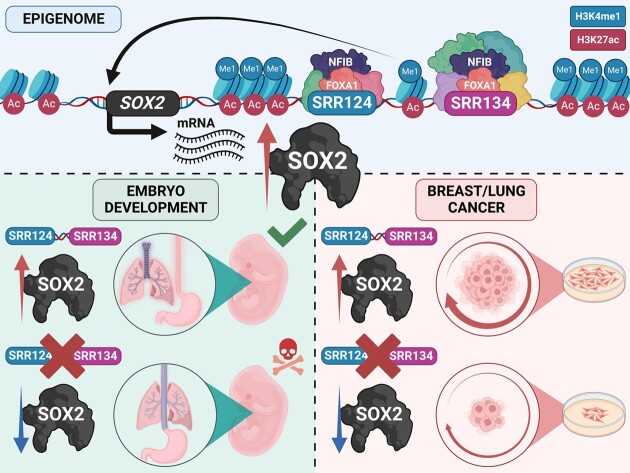

## INTRODUCTION

Developmental enhancers are commissioned during early embryogenesis, as transcription factors progressively restrict the epigenome through the repression of regulatory regions associated with pluripotency (1,2) and the activation of enhancers that control the expression of lineage-specific developmental genes (3–5). This establishes a cell type-specific epigenetic regulatory ‘memory’ that maintains cell lineage commitment and reinforces transcriptional programs (6). As cells mature and development ends, developmental-associated enhancers are decommissioned, and the enhancer landscape becomes highly restrictive and developmentally stable (6). This landscape, however, becomes profoundly disturbed during tumorigenesis, as cancer cells aberrantly acquire euchromatin features at regions near oncogenes (7,8) that are often associated with earlier stages of cell lineage specification (6). This ‘enhancer reprogramming’ has been proposed to result in a dysfunctional state that causes widespread abnormal gene expression and cellular plasticity (9–13). Although the misactivation of enhancers has been suggested as a major source of transcriptional dysregulation (reviewed in 14,15), it remains largely unclear how this mechanism unfolds during the progression of cancer. To study this process, we evaluated *cis-*regulatory elements involved in driving transcription during normal development and disease.

SRY-box transcription factor 2 (SOX2) is a pioneer transcription factor required for pluripotency maintenance in embryonic stem cells (16,17), involved in reprogramming differentiated cells to induced pluripotent stem cells in mammals (18–20), and acts as an oncogene in several different types of cancer (reviewed in 21,22). During later development, SOX2 is also required for tissue morphogenesis and homeostasis of the brain (23), eyes (24), esophagus (25), inner ear (26), lungs (27), skin (28), stomach (29), taste buds (30) and trachea (31) in both human and mouse. In these tissues, *SOX2* expression is regulated precisely in space and time at critical stages of development, although in most cases the *cis-*regulatory regions that mediate this precision remain unknown. For example, proper levels of *SOX2* expression are required during early development for the complete separation of the anterior foregut into the esophagus and trachea in mice (25,32,33) and in humans (34–36), as the disruption of *SOX2* expression leads to an abnormal developmental condition known as esophageal atresia with distal tracheoesophageal fistula (EA/TEF) (reviewed in 37,38). After the anterior foregut is properly separated in mice, *Sox2* expression ranges from the esophagus to the stomach in the gut (25,29), and throughout the trachea, bronchi and upper portion of the lungs in the developing airways (31). Proper branching morphogenesis at the tip of the lungs, however, requires temporary down-regulation of *Sox2*, followed by reactivation after lung bud establishment (27). *Sox2* also retains an essential function in multiple mature epithelial tissues, where it is highly expressed in proliferative and self-renewing adult stem cells necessary for replacing terminally differentiated cells within the epithelium of the brain, bronchi, esophagus, stomach and trachea (29,31,39,40). The expression of *Sox2*, however, becomes repressed as stem cells differentiate in these tissues (39).

As an oncogene, overexpression of *SOX2* is linked to increased cellular replication rates, aggressive tumor grades and poor patient outcomes in breast carcinoma (BRCA) (41–45), colon adenocarcinoma (COAD) (46–49), glioblastoma (GBM) (50–53), liver hepatocellular carcinoma (LIHC) (54), lung adenocarcinoma (LUAD) (55–57) and lung squamous cell carcinoma (LUSC) (58,59). These clinical and molecular characteristics arise from the participation of *SOX2* in the formation and maintenance of tumor-initiating cells that resemble tissue progenitor cells, as evidenced by BRCA (45,60,61), GBM (52,62–64), LUAD (65) and LUSC (66) studies. *SOX2* knockdown, on the other hand, often results in diminished levels of cell replication, invasion and treatment resistance in these tumor types (41,42,45,55,57,58,67–69). Despite the involvement of *SOX2* in the progression of multiple types of cancer, little is known about the mechanisms that cause *SOX2* overexpression during tumorigenesis. Two proximal enhancers were once deemed crucial for driving *Sox2* expression during early development: *Sox2* Regulatory Region 1 (SRR1) and SRR2 (23,70,71). Deletion of SRR1 and SRR2, however, has no effect on *Sox2* expression in mouse embryonic stem cells (72). In contrast, deletion of a distal *Sox2* Control Region (SCR), 106 kb downstream of the *Sox2* promoter, causes a profound loss of *Sox2* expression in mouse embryonic stem cells (72,73) and in blastocysts, where SCR deletion causes peri-implantation lethality (33). The contribution of these regulatory regions in driving *SOX2* expression during tumorigenesis, however, remains poorly defined.

Here, we investigated the mechanisms underlying *SOX2* overexpression in cancer. We found that, in breast and lung adenocarcinoma, *SOX2* is driven by a novel developmental enhancer cluster we termed SRR124–134, rather than the previously identified SRR1, SRR2 or the SCR. This novel distal cluster contains two regions located 124 and 134 kb downstream of the *SOX2* promoter that drive transcription in breast and lung adenocarcinoma cells. Deletion of this cluster results in significant *SOX2* down-regulation, leading to genome-wide changes in chromatin accessibility and a globally disrupted transcriptome. The SRR124–134 cluster is highly accessible in most breast and lung patient tumors, where chromatin accessibility at these regions is correlated with *SOX2* overexpression and is regulated positively by FOXA1 and negatively by NFIB. Finally, we found that both SRR124 and SRR134 are highly conserved in the mouse and are essential for postnatal survival, as homozygous deletion of their homologous regions results in lethal EA/TEF. These findings serve as a prime example of how different types of cancer cells reprogram enhancers that were decommissioned during development to drive the expression of oncogenes during tumorigenesis.

MATERIALS AND METHODS

#### Cell culture

MCF-7 cells were obtained from Eldad Zacksenhaus (Toronto General Hospital Research Institute, Toronto, ON, Canada). H520 (HTB-182) and T47D (HTB-133) cells were acquired from the ATCC. PC-9 (90071810) cells were obtained from Sigma. Cell line identities were confirmed by short tandem repeat profiling. MCF-7 and T47D cells were grown in phenol red-free Dulbecco’s modified Eagle’s medium (DMEM) high glucose (Gibco), 10% fetal bovine serum (FBS) (Gibco), 1× Glutamax (Gibco), 1× sodium pyruvate (Gibco), 1× penicillin–streptomycin (Gibco), 1× non-essential amino acids (Gibco), 25 mM HEPES (Gibco) and 0.01 mg/ml insulin (Sigma). H520 and PC-9 cells were grown in phenol red-free RPMI-1640 (Gibco), 10% FBS (Gibco), 1× Glutamax (Gibco), 1× sodium pyruvate (Gibco), 1× penicillin–streptomycin (Gibco), 1× non-essential amino acids (Gibco) and 25 mM HEPES (Gibco). Cells were either passaged or had their medium replenished every 3 days.

#### Genome editing

Guide RNA (gRNA) sequences were designed using Benchling. We minimized the possibility of unwanted off-target mutations by strictly selecting gRNA with no off-target sites with <3 bp mismatches. Pairs of gRNA plasmids were constructed by inserting a 20 bp target sequence (Supplementary Table S1) into an empty gRNA cloning vector (a gift from George Church; Addgene plasmid #41824) (74) containing either miRFP670 (Addgene plasmid #163748) or tagBFP (Addgene plasmid #163747) fluorescent markers. Plasmids were sequenced to confirm correct insertion. Both gRNA (1 μg each) vectors were co-transfected with 3 μg of pCas9_GFP (a gift from Kiran Musunuru; Addgene plasmid #44719) (75) using Neon electroporation (Life Technologies). After 72 h of transfection, cells were sorted by fluorescence-activated cell sorting (FACS) to select clones that contained all three plasmids. Sorted tagBFP^+^/GFP^+^/miRFP670^+^ cells were grown in a bulk population and serially diluted into individual wells to generate isogenic populations. Once fully grown, each well was screened by polymerase chain reaction (PCR) to confirm the deletion (Supplementary Table S2). Enhancer-deleted cells are available to the research community upon request.

#### Gene tagging

*SOX2* was tagged with a P2A-tagBFP sequence in both alleles using clustered regularly interspaced palindromic repeats (CRISPR)-mediated homology-directed repair (HDR) (76). This strategy results in the expression of a single transcript that is further translated into two separate proteins due to ribosomal skipping (77). In summary, we designed a gRNA that targets the 3′ end of the *SOX2* stop codon (Supplementary Table S1, Addgene plasmid #163752). We then amplified ∼800 bp homology arms upstream and downstream of the gRNA target sequence using high-fidelity Phusion Polymerase. We purposely avoided amplification of the *SOX2* promoter sequence to reduce the likelihood of random integrations in the genome. Both homology arms were then joined at each end of a P2A-tagBFP sequence using Gibson assembly. Flanking primers containing the gRNA target sequence were used to reamplify *SOX2*-P2A-tagBFP and add gRNA targets at both ends of the fragment; this approach allows excision of the HDR sequence from the backbone plasmid once inside the cell (78). Finally, the full HDR sequence was inserted into a pJET1.2 (Thermo Scientific) backbone, midiprepped and sequenced (Addgene #163751). A 3 μg aliquot of HDR template was then co-transfected with 1 μg of hCas9 (a gift from George Church; Addgene plasmid #41815) (74) and 1 μg of gRNA plasmid using Neon electroporation (Life Technologies). A week after transfection, tagBFP^+^ cells were FACS sorted as a bulk population. Sorted cells were further grown for 2 weeks, and single tagBFP^+^ cells were isolated to generate isogenic populations. Once fully grown, each clone was screened by PCR and sequenced to confirm homozygous integration of P2A-tagBFP into the *SOX2* locus (Supplementary Table S2). MCF-7 *SOX2*-P2A-tagBFP cells are available to the research community upon request.

#### Luciferase assay

Luciferase activity was measured using the dual-luciferase reporter assay (Promega #E1960) that relies on the co-transfection of two plasmids: pGL4.23 (firefly luciferase, *luc2*) and pGL4.75 (*Renilla* luciferase). Assayed plasmids were constructed by subcloning the empty pGL4.23 vector containing a minimal promoter (minP). SRR124, SRR134, SRR1, SRR2 and hSCR were PCR amplified (primers are given in Supplementary Table S3) from MCF-7 genomic DNA using high-fidelity Phusion Polymerase and inserted in the forward position downstream of the *luc2* gene at the NotI restriction site. Constructs were sequenced to confirm correct insertions.

JASPAR2022 (79) was used to detect FOXA1 (GTAAACA) and NFIB (TGGCAnnnnGCCAA) motifs in the SRR134 sequence. Only motifs with a score of ≥80% were further analyzed. Bases within each motif sequence were mutated until the score was reduced below 80% without affecting co-occurring motifs or creating novel binding sites. In total, four FOXA1 motifs and two NFIB motifs were mutated (Supplementary Table S4). Engineered sequences were ordered as gene blocks (Eurofins) and inserted into pGL4.23 in the forward position. Constructs were sequenced to confirm correct insertions.

Cells were plated in 96-well plates with four technical replicates at 2 × 10^4^ cells per well. After 24 h, a 200 ng 50:1 mixture of enhancer vector and pGL4.75 was transfected using Lipofectamine 3000 (0.05 μl of Lipofectamine:1 μl of Opti-mem). For transcription factor overexpression analysis, a 200 ng 50:10:1 mixture of enhancer vector, expression plasmid and pGL4.75 was transfected. After 48 h of transfection, cells were lysed in 1× Passive Lysis Buffer and stored at –80°C until all five biological replicates were completed. Luciferase activity was measured in the Fluoroskan Ascent FL plate reader. Enhancer activity was calculated by normalizing the firefly signal from pGL4.23 to the *Renilla* signal from pGL4.75.

#### Colony formation assay

MCF-7 and PC-9 cells were seeded at low density (2,000 cells/well) into 6-well plates in triplicate for each cell line. Culture medium was renewed every 3 days. After 12 days, cells were fixed with 3.7% paraformaldehyde for 10 min and stained with 0.5% crystal violet for 20 min to quantify the number of colonies formed. Crystal violet staining was then eluted with 10% acetic acid and absorbance was measured at 570 nm to evaluate cell proliferation. Each 6-well plate was considered one biological replicate and the experiment was repeated five times for each cell line (*n* = 5).

#### FACS analysis

For analyzing the effects of *FOXA1* and *NFIB* overexpression, 2 × 10^6^ SOX2-P2A-tagBFP cells were transfected with 50 nM of plasmid expressing either miRFP670 (a gift from Vladislav Verkhusha; Addgene plasmid #79987), FOXA1-T2A-miRFP670 (Addgene plasmid #182335) or NFIB-T2A-miRFP670 (Addgene plasmid #187222) in five replicates. Five days after transfection, miRFP670, tagBFP and propidium iodide (PI) (live/dead stain) signals were acquired using FACS; the amount of tagBFP signal from miRFP670^+^/PI^–^ cells was compared between each treatment across all replicates.

FlowJo's chi-squared T(x) test was used to compare the effects of each treatment on tagBFP expression; T(x) scores >1000 were considered ‘strongly significant’ (***), whereas T(x) scores <100 were considered ‘non-significant’.

#### Transcriptome analysis

Total RNA was isolated from wild-type (WT; ΔENH^+/+^) and enhancer-deleted (ΔENH^–/–^) cell lines using the RNeasy kit. Genomic DNA was digested by Turbo DNase. A 500–2,000 ng aliquot of total RNA was used in a reverse transcription reaction with random primers. cDNA was diluted in H_2_O and amplified in a quantitative PCR (qPCR) using SYBR Select Mix (primers are given in Supplementary Table S5). Amplicons were sequenced to confirm primer specificity. Gene expression was normalized to *PUM1* (80–82).

Total RNA was sent to The Centre for Applied Genomics (TCAG) for paired-end rRNA-depleted total RNA-seq (Illumina 2500, 125 bp). Read quality was checked by fastQC, trimmed using fastP (83) and mapped to the human genome (GRCh38/hg38) using STAR 2.7 (84). Normal breast epithelium RNA-seq was obtained from ENCODE (Supplementary Table S6) (85,86). Mapped reads were quantified using featureCounts (87) and imported into DESeq2 (88) for normalization and differential expression analysis. Genes with a |log_2_ fold change (FC)| > 1 and false discovery rate (FDR)-adjusted *Q* < 0.01 were considered significantly changed. Differential gene expression was plotted using the EnhancedVolcano package. Correlation and clustering heatmaps were plotted using the pheatmap R package (https://cran.r-project.org/web/packages/pheatmap/index.html). A signal enrichment plot was prepared using NGS.plot (89).

Cancer patient transcriptome data were obtained from TCGA (90) using the TCGAbiolinks package (91). The overall survival KM-plot (92) was calculated using clinical information from TCGA (93). Tumor transcriptome data were compared with normal tissue using DESeq2. RNA-seq reads were normalized to library size using DESeq2 (88) and transformed to a log_2_ scale [log_2_ counts]. Differential gene expression was considered significant if |log_2_ FC| > 1 and *Q* < 0.01.

Gene set enrichment analysis (GSEA) was performed by ranking genes according to their log_2_ FC in ΔENH^–/–^ versus ΔENH^+/+^ MCF-7 cells. The ranking was then analyzed using the GSEA function from the clusterProfiler package (94) with a threshold of FDR-adjusted *Q* < 0.05 using the MSigDB GO term database (C5).

#### Chromatin accessibility analysis

Cells were grown in three separate wells (*n* = 3) and 50,000 cells were sent to the Princess Margaret Genomics Centre for ATAC-seq library preparation using the Omni-ATAC protocol (95). ATAC-seq libraries were sequenced using 50 bp paired-ended parameters in the Illumina Novaseq 6000 platform. Read quality was checked by fastQC, trimmed using fastP and mapped to the human genome (GRCh38/hg38) using STAR 2.7. Narrow peaks were called using Genrich (https://github.com/jsh58/Genrich). Differential chromatin accessibility analysis was performed using diffBind (96). ATAC-seq peaks with a |log_2_ FC| > 1 and FDR-adjusted *Q* < 0.01 were considered significantly changed. Correlation heatmaps were generated using diffBind. A signal enrichment plot was prepared using NGS.plot (89). Genes were separated into three categories according to their expression levels in our ΔENH^+/+^ MCF-7 RNA-seq data.

Transcription factor footprint analysis was performed using TOBIAS (97) with standard settings. Motifs with a |log_2_ FC| > 0.1 and FDR-adjusted *Q* < 0.01 were considered significantly enriched in each condition. Replicates (*n* = 3) were merged into a single BAM file for each condition. Motif enrichment at differential ATAC-seq peaks was performed using HOMER (98). ATAC-seq peaks were assigned to their closest gene within ± 1 Mb distance from their promoter using ChIPpeakAnno (99).

Cancer patient ATAC-seq data were obtained from TCGA (100). DNase-seq data from human developing tissues were obtained from ENCODE (Supplementary Table S6) (85,86). Read quantification was calculated at the *RAB7a* (pRAB7a), *OR5K1* (pOR5K1) and *SOX2* (pSOX2) promoters, together with SRR1, SRR2, SRR124, SRR134, hSCR and desert regions with a 1500 bp window centered at the core of each region (genomic coordinates of each region are given in Supplementary Table S7). Reads were normalized to library size [reads per million (RPM)] and transformed to a log_2_ scale (log_2_ RPM) using a custom script (https://github.com/luisabatti/BAMquantify). Each region's average log_2_ RPM was compared with that of the *OR5K1* promoter for differential analysis using Dunn's test with Holm correction. Correlations were calculated using Pearson's correlation test and considered significant if FDR-adjusted *Q* < 0.05. Chromatin accessibility at SRR124 and SRR134 regions was considered low if log_2_ RPM < –1, medium if –1 ≤ log_2_ RPM ≤ 1 or high if log_2_ RPM > 1.

ATAC-seq data from developing mouse lung and stomach tissues were obtained from ENCODE (Supplementary Table S6) (85) and others (101). Conserved mouse regulatory regions were lifted from the human build (GRCh38/hg38) to the mouse build (GRCm38/mm10) using UCSC liftOver (102). The number of mapped reads was calculated at the *Egf* (pEgf), *Olfr266* (pOlfr266) and *Sox2* (pSox2) promoters, together with the mouse mSRR1, mSRR2, mSRR96, mSRR102, mSCR and desert regions with a 1500 bp window at each location (genomic coordinates are given in Supplementary Table S8). Each log_2_-transformed region's RPM (log_2_ RPM) was compared with that of the negative *Olfr266* promoter control for differential analysis using Dunn's test with Holm correction.

#### Conservation analysis

Cross-species evolutionary conservation was obtained using phyloP (103). Pairwise comparisons between human SRR124 and SRR134 (GRCh38/hg38) and mouse mSRR96 and mSRR102 (GRCm38/mm10) sequences were aligned using Clustal Omega (104) and plotted using FlexiDot (105) with an 80% conservation threshold.

#### ChIP-seq analysis

ChIP-seq data for transcription factor and histone modifications were obtained from ENCODE (85) (Supplementary Table S6) and others (106–108) (Supplementary Table S9). H3K4me1 and H3K27ac tracks were normalized to input and library size (log_2_ RPM). Histone modification ChIP-seq tracks and transcription factor ChIP-seq peaks were uploaded to the UCSC browser (102) for visualization. Normalized H3K4me1 and H3K27ac reads were quantified and the difference in normalized signal was calculated using diffBind. Peaks with a |log_2_ FC| > 1 and *Q* < 0.01 were considered significantly changed.

Overlapping ChIP-seq and ATAC-seq peaks were analyzed using ChIPpeakAnno (99). The hypergeometric test was performed by comparing the number of overlapping peaks with the total size of the genome divided by the median peak size.

#### Mouse line construction

Our mSRR96–102 knockout mouse line (C57BL/6J; Chr3_SRR124-SRR134_del) was ordered from and generated by The Centre for Phenogenomics (TCP) model production core in Toronto, ON. The protocol for the generation of the mouse line has been previously described (109). Briefly, C57BL/6J zygotes were collected from superovulated, mated and plugged female mice at 0.5 days post-coitum. Zygotes were electroporated with CRISPR-associated protein 9 (Cas9) ribonucleoprotein (RNP) complexes (gRNA sequences are given in Supplementary Table S1) and transferred into pseudopregnant female recipients within 3–4 hours of electroporation. Newborn pups (potential founders) were screened by endpoint PCR and sequenced to confirm allelic mSRR96–102 deletions (Supplementary Table S2). One heterozygous mSRR96–102 founder (ΔmENH^+/–^) was then backcrossed twice to the parental strain to reduce the probability of off-target mutation segregation and to confirm germline transmission. Off-target mutagenesis by Cas9 is rare in mouse embryos using this protocol (110). Neither of the two gRNAs used for the mSRR96–102 deletion had any predicted off-target sites with <3 bp mismatches. Furthermore, no off-target hits were found within exonic regions on chromosome 3, where *Sox2* is located. Potential changes in chromosomal copy numbers were also ruled out by real-time PCR.

Once the mouse line was established and the mSRR96–102 deletion was fully confirmed and sequenced in the N1 offspring, ΔmENH^+/–^ mice were crossed and the number of live pups from each genotype (ΔmENH^+/+^, ΔmENH^+/–^, ΔmENH^–/–^) was assessed at weaning (P21). The obtained number of live pups from each genotype was then compared with the expected Mendelian ratio of 1:2:1 (ΔmENH^+/+^:ΔmENH^+/–^:ΔmENH^–/–^) using a chi-squared test. Once the lethality of the homozygous deletion was confirmed at weaning, E18.5 littermate embryos generated from new ΔmENH^+/–^ crosses were collected for further histological analyses.

All procedures involving animals were performed in compliance with the Animals for Research Act of Ontario and the Guidelines of the Canadian Council on Animal Care. The TCP Animal Care Committee reviewed and approved all procedures conducted on animals at the facility. Sperm from male ΔmENH^+/–^ mice has been cryopreserved at the Canadian Mouse Mutant Repository (CMMR) and is available upon request.

#### Histological analyses

A total of 46 embryos were collected at E18.5 and fixed in 4% paraformaldehyde. Each of these embryos was genotyped. A total of 15 embryos (Supplementary Table S10), five of each genotype (ΔmENH^+/+^, ΔmENH^+/–^, ΔmENH^–/–^), were randomly selected, processed and embedded in paraffin for sectioning and further analysis. Tissue sections were collected at 4 μm thickness roughly at the start of the thymus. Sections were prepared by the Pathology Core at TCP.

Tissue sections were stained with hematoxylin and eosin (H&E) using an auto-stainer to ensure batch consistency. Slides were scanned using a Hamamatsu Nanozoomer slide scanner at ×20 magnification. For immunohistochemistry staining, E18.5 embryo cross-sections were submitted to heat-induced epitope retrieval with Tris-EDTA (pH 9.0) for 10 min, followed by quenching of endogenous peroxidase with Bloxall reagent (Vector). Non-specific antibody binding was blocked with 2.5% normal horse serum (Vector), followed by incubation for 1 hour in rabbit anti-SOX2 (Abcam, ab92494, 1:500). After washes, sections were incubated for 30 min with ImmPRESS anti-rabbit horseradish peroxidase (HRP; Vector), followed by 3,3′-diaminobenzidine (DAB) reagent and counterstained in Mayer's hematoxylin.

For immunofluorescence staining, E18.5 embryo cross-sections were collected onto charged slides and then baked at 60°C for 30 min. Tissue sections were submitted to heat-induced epitope retrieval with citrate buffer pH 6.0 for 10 min. Non-specific antibody binding was blocked with Protein Block Serum-Free (Dako) for 10 min, followed by overnight incubation at 4°C in a primary antibody cocktail (rabbit anti-NKX2.1, Abcam ab76013 at 1:200; rat anti-SOX2, Thermo Fisher Scientific 14–9811-80 at 1:100). After washes with TBS-T, sections were incubated for 1 hour with a cocktail of Alexa Fluor-conjugated secondary antibodies at 1:200 (goat anti-rabbit IgG AF488, Thermo Fisher Scientific A32731; goat anti-rat IgG AF647, Thermo Fisher Scientific A21247), followed by counterstaining with 4′,6-diamidino-2-phenylindole (DAPI). Scanning was performed using an Olympus VS-120 slide scanner and imaged using a Hamamatsu ORCA-R2 C10600 digital camera for all dark-field and fluorescent images.

## RESULTS

### Two regions downstream of *SOX2* gain enhancer features in cancer cells

**Figure 1.**
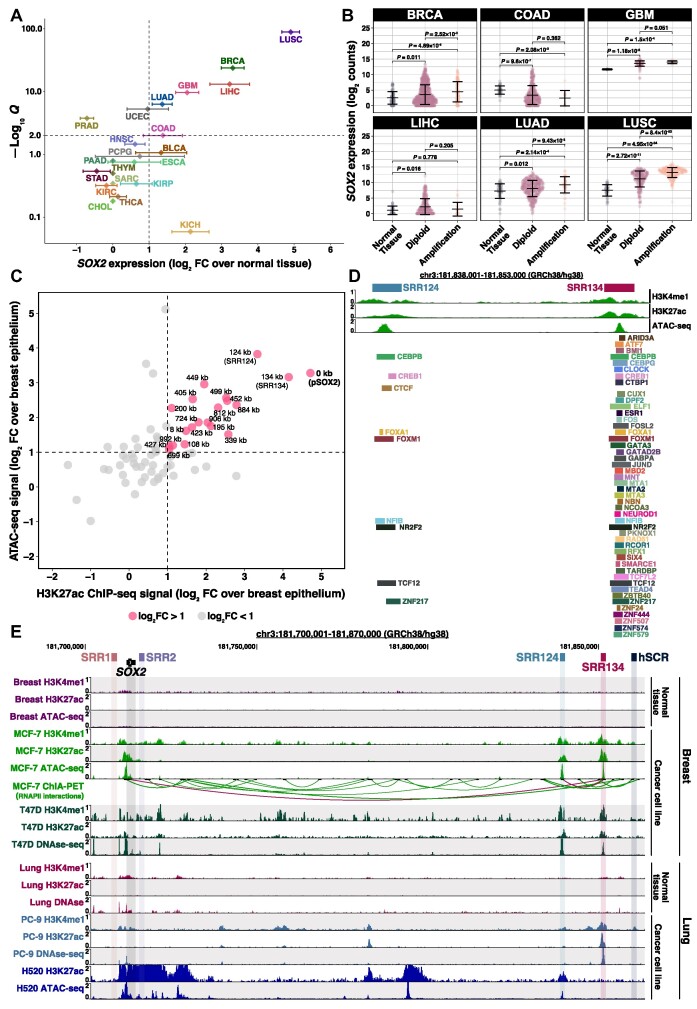
A cluster 124–134 kb downstream of *SOX2* gains enhancer features in cancer cells. (**A**) Super-logarithmic RNA-seq volcano plot of *SOX2* expression from 21 cancer types compared with normal tissue (90). Cancer types with log_2_ FC > 1 and FDR-adjusted *Q*< 0.01 were considered to significantly overexpress *SOX2*. Error bars: standard deviation (SD). (**B**) *SOX2* log_2_-normalized expression (log_2_ counts) associated with the *SOX2* copy number from BRCA (*n* = 1174), COAD (*n* = 483), GBM (*n* = 155), LIHC (*n* = 414), LUAD (*n* = 552) and LUSC (*n* = 546) patient tumors (90). RNA-seq reads were normalized to library size using DESeq2 (88). Error bars: SD. Significance analysis by Dunn's test (180) with Holm correction (181). (**C**) 1500 bp genomic regions within ± 1 Mb from the *SOX2* transcription start site (TSS) that gained enhancer features in MCF-7 cells (85) compared with normal breast epithelium (86). Regions that gained both ATAC-seq and H3K27ac ChIP-seq signal above our threshold (log_2_ FC > 1, dashed line) are highlighted in pink. Each region was labeled according to their distance in kilobases to the *SOX2* promoter (pSOX2, bold). (**D**) ChIP-seq signal for H3K4me1 and H3K27ac, ATAC-seq signal and transcription factor ChIP-seq peaks at the SRR124–134 cluster in MCF-7 cells. Datasets are from ENCODE (85). (**E**) UCSC Genome Browser (102) display of H3K4me1 and H3K27ac ChIP-seq signal, DNase-seq and ATAC-seq chromatin accessibility signal, and ChIA-PET RNA polymerase II (RNAPII) interactions around the *SOX2* gene within breast (normal tissue and 2 BRCA cancer cell lines) and lung (normal tissue, one LUAD and one LUSC cancer cell line) samples (85,106,^108^,^127^). Relevant RNAPII interactions (between SRR124 and SRR134, and between SRR134 and pSOX2) are highlighted in maroon.

*SOX2* overexpression occurs in multiple types of cancer (reviewed in 21,22). To examine which cancer types have the highest levels of *SOX2* up-regulation, we performed differential expression analysis by calculating the log_2_ FC of *SOX2* transcription from 21 TCGA primary solid tumors (see Supplementary Table S11 for cancer type abbreviations) compared with normal tissue samples (90). We found that BRCA (log_2_ FC = 3.31), COAD (log_2_ FC = 1.38), GBM (log_2_ FC = 2.05), LIHC (log_2_ FC = 3.22), LUAD (log_2_ FC = 1.36) and LUSC (log_2_ FC = 4.91) tumors had the greatest *SOX2* up-regulation (log_2_ FC > 1; FDR-adjusted *Q* < 0.01; Figure 1A; Supplementary Table S12). As a negative control, we ran this same analysis using the housekeeping gene *PUM1* (81) and found no cancer types with significant up-regulation of this gene (Supplementary Figure S1A; Supplementary Table S13).

Next, we divided BRCA, COAD, GBM, LIHC, LUAD and LUSC patients (*n* = 3064) into four groups according to their *SOX2* expression. Gene expression levels were measured by RNA-seq counts normalized to library size and transformed to a log_2_ scale, hereinafter referred to as log_2_ counts. Cancer patients within the top group (25% highest *SOX2* expression; log_2_ counts > 10.06) have a significantly (*P* = 1.27 × 10^−23^, log-rank test) lower overall probability of survival compared with cancer patients within the bottom group (25% lowest *SOX2* expression; log_2_ counts < 1.68) (Supplementary Figure S1B; Supplementary Table S14). We also examined the relationship between *SOX2* copy number and *SOX2* overexpression within these six tumor types. Although previous studies have shown that *SOX2* is frequently amplified in squamous cell carcinoma (58,59,111,112), we found that most BRCA (88%), COAD (98%), GBM (91%), LIHC (94%) and LUAD (92%) tumors were diploid for *SOX2*. In addition, BRCA (*P* = 0.011, Holm-adjusted Dunn's test), GBM (*P* = 1.18 × 10^−3^), LIHC (*P* = 0.016), LUAD (*P* = 0.012) and LUSC (*P* = 2.72 × 10^−11^) diploid tumors significantly overexpressed *SOX2* compared with normal tissue (Figure 1B; Supplementary Table S15). This indicates that gene amplification is dispensable for driving *SOX2* overexpression in most cancer types.

We investigated whether the *SOX2* locus gains epigenetic features associated with active enhancers in cancer cells. Enhancer features commonly include accessible chromatin determined by either Assay for Transposase Accessible Chromatin with high-throughput sequencing (ATAC-seq) (113) or DNase I-hypersensitive sites sequencing (DNase-seq) (114), and histone modifications including histone H3 lysine 4 monomethylation (H3K4me1) and histone H3 lysine 27 acetylation (H3K27ac) (115,116). To study gains in enhancer features within the *SOX2* locus, we initially focused our analyses on luminal A breast cancer, the most common subtype of BRCA to significantly (*P* = 0.021, Tukey's test) overexpress *SOX2* (Supplementary Figure S1C) (90,117). MCF-7 cells are a widely used ER^+^/PR^+^/HER2^−^ luminal A breast adenocarcinoma model (118), which have been previously described to overexpress *SOX2* (41,69,119,120). After confirming that *SOX2* is one of the most up-regulated genes in MCF-7 cells (log_2_ FC = 10.75; FDR-adjusted *Q* = 2.20 × 10^−36^; Supplementary Figure S1D; Supplementary Table S16) compared with normal breast epithelium (86), we contrasted their chromatin accessibility and histone modifications (85). By intersecting 1500 bp regions that contain at least a 500 bp overlap between H3K27ac and ATAC-seq peaks, we found that 19 putative enhancers gained (log_2_ FC > 1) both these features within ± 1 Mb from the *SOX2* transcription start site (TSS) in MCF-7 cells (Figure 1C; Supplementary Table S17). Besides the *SOX2* promoter (pSOX2), we identified a downstream cluster containing two regions that have gained the highest ATAC-seq and H3K27ac signal in MCF-7 cells: SRR124 (124 kb downstream of pSOX2) and SRR134 (134 kb downstream of pSOX2). The previously described SRR1, SRR2 (23,70,71) and hSCR (72,73), however, lacked substantial gains in enhancer features within MCF-7 cells.

Alongside gains in chromatin features, another characteristic of active enhancers is the binding of numerous (> 10) transcription factors (121–123). Chromatin immunoprecipitation sequencing (ChIP-seq) data from ENCODE (85) on 117 transcription factors revealed 48 different factors present at the SRR124–134 cluster in MCF-7 cells, with the majority (47) of these factors present at SRR134 (Figure 1D). Transcription factors bound at both SRR124 and SRR134 include CEBPB, CREB1, FOXA1, FOXM1, NFIB, NR2F2, TCF12 and ZNF217. An additional feature of distal enhancers is that they contact their target genes through long-range chromatin interactions (124,125). We analyzed Chromatin Interaction Analysis by Paired-End-Tag sequencing (ChIA-PET) data from MCF-7 cells (126) and found two interesting RNA polymerase II (RNAPII)-mediated chromatin interactions: one between the *SOX2* gene and SRR134, and one between SRR124 and SRR134 (Figure 1E). Beyond the interactions with SOX2, we also identified long-range interactions between SRR124 and the upstream long non-coding RNA (lncRNA) *SOX2-OT* (∼665 kb away), between SRR134 and the downstream lncRNA *LINC01206* (∼150 kb away), and between SRR134 and the upstream *RSRC1* gene (∼23 Mb away) (Supplementary Table S18). In addition to MCF-7 cells, we found that H520 (LUSC), PC-9 (LUAD) and T47D (luminal A BRCA) cancer cell lines, which display varying levels of *SOX2* expression (Supplementary Figure S1E), also gained substantial enhancer features at SRR124 and SRR134 when compared with normal tissue (Figure 1E) (85,106,108,127). Together, these data suggest that SRR124 and SRR134 could be active enhancers driving *SOX2* transcription in BRCA, LUAD and LUSC.

### The SRR124–134 cluster is essential for *SOX2* expression in BRCA and LUAD cells

**Figure 2.**
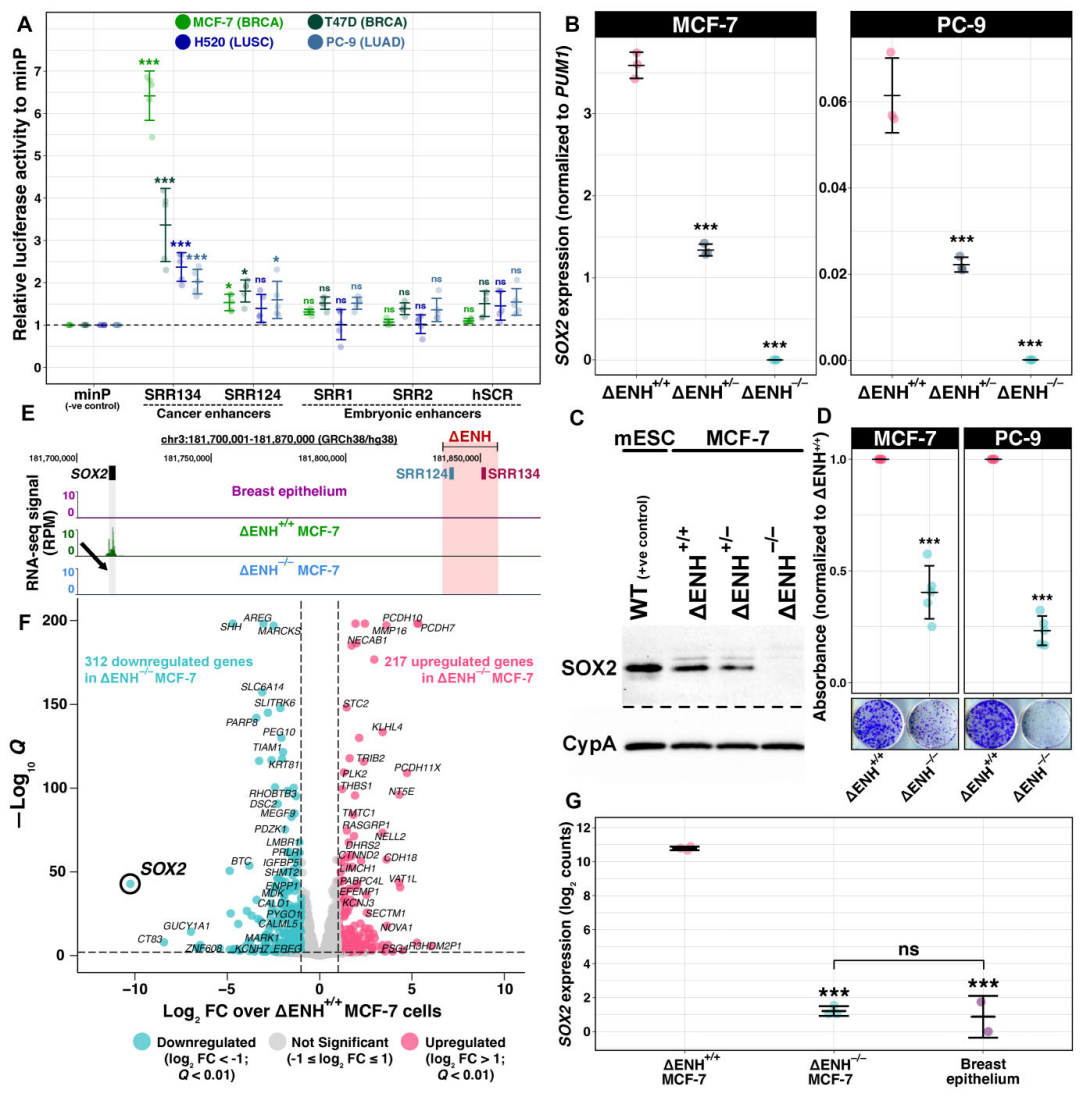
The SRR124–134 cluster drives *SOX2* overexpression in BRCA and LUAD cells. (**A**) Enhancer reporter assay comparing luciferase activity driven by the SRR1, SRR2, SRR124, SRR134 and hSCR regions with an empty vector containing only a minimal promoter (minP). Enhancer constructs were assayed in the BRCA (MCF-7, T47D), LUAD (PC-9) and LUSC (H520) cell lines. Dashed line: average activity of minP. Error bars: SD. Significance analysis by Dunnett's test (*n* = 5; *P < 0.05, ***P < 0.001, ns: not significant) (182). (**B**) RT–qPCR analysis of *SOX2* transcript levels in SRR124–134 heterozygous- (ΔENH^+/–^) and homozygous- (ΔENH^–/–^) deleted MCF-7 (BRCA) and PC-9 (LUAD) clones compared with WT (ΔENH^+/+^) cells. Error bars: SD. Significance analysis by Dunnett's test (*n* = 3; ***P < 0.001). (**C**) SOX2 protein levels in mouse embryonic stem cells (mESCs, positive control), ΔENH^+/+^, ΔENH^+/–^ and ΔENH^–/–^ MCF-7 clones. Cyclophilin A (CypA) was used as a loading control across all samples. (**D**) Colony formation assay with ΔENH^+/+^ and ΔENH^–/–^ MCF-7 and PC-9 cells. Total crystal violet absorbance was normalized relative to the average absorbance from ΔENH^+/+^ cells for each respective cell line. Significance analysis by *t*-test with Holm correction (*n* = 5; ***P < 0.001). (**E**) UCSC Genome Browser (102) view of the SRR124–134 cluster deletion in ΔENH^–/–^ MCF-7 cells with RNA-seq tracks from normal breast epithelium (86), ΔENH^+/+^ and ΔENH^–/–^ MCF-7 cells. Arrow: reduction in RNA-seq signal at the *SOX2* gene in ΔENH^–/–^ MCF-7 cells. (**F**) Volcano plot with DESeq2 (88) differential expression analysis between ΔENH^–/–^ and ΔENH^+/+^ MCF-7 cells. Blue: 312 genes that significantly lost expression (log_2_ FC < –1; FDR-adjusted *Q* < 0.01) in ΔENH^–/–^ MCF-7 cells. Pink: 217 genes that significantly gained expression (log_2_ FC > 1; *Q* < 0.01) in ΔENH^–/–^ MCF-7 cells. Gray: 35 891 genes that maintained similar (–1 ≤ log_2_ FC ≤ 1) expression between ΔENH^–/–^ and ΔENH^+/+^ MCF-7 cells. (**G**) Comparison of *SOX2* transcript levels between ΔENH^+/+^ and either ΔENH^–/–^ MCF-7 or normal breast epithelium cells (86), and between ΔENH^–/–^ MCF-7 and normal breast epithelium cells. RNA-seq reads were normalized to library size using DESeq2 (88). Error bars: SD. Significance analysis by Tukey's test (***P < 0.001, ns: not significant) (183).

To assess SRR124 and SRR134 enhancer activity alongside the embryonic-associated SRR1, SRR2 and hSCR regions, we used a reporter vector containing the firefly luciferase gene under the control of a minimal promoter (minP, pGL4.23). We transfected each enhancer construct into the BRCA (MCF-7, T47D), LUAD (PC-9) and LUSC (H520) cell lines and measured luciferase activity as a relative FC compared with the empty minP vector. SRR134 demonstrated the strongest enhancer activity, with the MCF-7 (FC = 6.42; *P* < 2 × 10^−16^, Dunnett's test), T47D (FC = 3.36; *P* = 9.34 × 10^−10^), H520 (FC = 2.37; *P* = 1.22 × 10^−6^) and PC-9 (FC = 2.03; *P* = 9.79 × 10^−5^) cell lines displaying a significant increase in luciferase activity compared with minP (Figure 2A). SRR124 also showed a modest, significant increase in luciferase activity compared with minP in the MCF-7 (FC = 1.53; *P* = 4.27 × 10^−2^), T47D (FC = 1.80; *P* = 4.57 × 10^−2^) and PC-9 (FC = 1.60; *P* = 4.27 × 10^−2^) cell lines. The SRR1, SRR2 and hSCR enhancers, however, showed no significant enhancer activity (*P* > 0.05) in any of the four cell lines.

Although reporter assays can be used to assess enhancer activity, enhancer knockout approaches remain the current gold standard method for enhancer validation (128,129). To investigate whether the SRR124–134 cluster drives *SOX2* expression in cancer cells, we used CRISPR/Cas9 to delete this cluster from breast (MCF-7, T47D) and lung (H520, PC-9) cancer cell lines (Supplementary Figure S2A). Reverse transcription–qPCR (RT–qPCR) showed that homozygous SRR124–134 deletion (ΔENH^–/–^) causes a profound (> 99.5%) and significant (*P* < 0.001, Dunnett's test) loss of *SOX2* expression compared with non-deleted cells (ΔENH^+/+^) in both the MCF-7 and PC-9 cell lines (Figure 2B). Heterozygous SRR124–134 deletion (ΔENH^+/–^) also significantly (*P* < 0.001) reduced *SOX2* expression by ∼60% in both MCF-7 and PC-9 cells (Figure 2B). Immunoblot analysis confirmed the depletion of the SOX2 protein in ΔENH^–/–^ MCF-7 cells (Figure 2C). Although we were unable to isolate a homozygous deletion clone from T47D cells, multiple independent heterozygous ΔENH^+/–^ T47D clonal isolates also showed a significant down-regulation (>50%; *P* < 0.001) in *SOX2* expression (Supplementary Figure S2B). H520 cells, on the other hand, showed no significant (*P* > 0.05) impact on *SOX2* expression following either heterozygous or homozygous deletions (Supplementary Figure S2C), which indicates that *SOX2* transcription is sustained by a different mechanism in these cells. To assess the impact of the loss of *SOX2* expression in the tumor initiation capacity of enhancer-deleted cells, we performed a colony formation assay with MCF-7 and PC-9 ΔENH^–/–^ cells. We found that both MCF-7 (*P* = 3.53 × 10^−4^, *t*-test) and PC-9 (*P* = 1.26 × 10^−5^) ΔENH^–/–^ cells showed a significant decrease (> 50%) in their ability to form colonies compared with ΔENH^+/+^ cells (Figure 2D), further underscoring the crucial role of SRR124–134-driven *SOX2* overexpression in sustaining the elevated tumor initiation potential in both BRCA and LUAD.

Next, we performed total RNA sequencing (RNA-seq) to measure changes in the transcriptome of ΔENH^–/–^ MCF-7 cells compared with ΔENH^+/+^ MCF-7 cells. Although RNA-seq mainly measures the steady-state level of RNA molecules in the cell, we opted for this approach to provide a broad perspective on the transcriptional changes resulting from the SRR124–134 deletion and to detect any *SOX2* transcripts if they were present. As expected, all three replicates of each genotype clustered together (Supplementary Figure S2D). In addition to *SOX2* down-regulation (Figure 2E), differential expression analysis showed a total of 529 genes differentially (|log_2_ FC| > 1; FDR-adjusted *Q* < 0.01) expressed in ΔENH^–/–^ MCF-7 cells (Figure 2F; Supplementary Table S19). From these, 312 genes significantly lost expression (59%), whereas 217 (41%) genes significantly gained expression in ΔENH^–/–^ compared with ΔENH^+/+^ MCF-7 cells (Supplementary Figure S2E). *SOX2* was the gene with the greatest loss in expression (log_2_ FC = –10.24; *Q* = 1.23 × 10^−43^) in ΔENH^–/–^ MCF-7 cells, followed by *CT83* (log_2_ FC = –8.43; *Q* = 1.07 × 10^−8^) and *GUCY1A1* (log_2_ FC = –6.96; *Q* = 5.09 × 10^−15^). Interestingly, the expression of the lncRNA *SOX2-OT* was also significantly down-regulated (log_2_ FC = –2.23; *Q* = 4.64 × 10^−4^) in ΔENH^–/–^ MCF-7 cells (Supplementary Table S19). However, since this transcript overlaps the *SOX2* coding region, it is unclear if this reduction is a direct result of the SRR124–134 deletion or secondary to *SOX2* down-regulation. Despite showing chromatin interactions with the SRR124–134 cluster, transcription of the *RSRC1* gene and the lncRNA *LINC01206* remained unchanged (*Q* > 0.05) in ΔENH^–/–^ MCF-7 cells. Genes with the most substantial gains in expression within ΔENH^–/–^ MCF-7 cells included the protocadherins *PCDH7* (log_2_ FC = 5.34; *Q* < 1 × 10^−200^), *PCDH10* (log_2_ FC = 5.29; *Q* < 1 × 10^−200^) and *PCDH11X* (log_2_ FC = 4.73; Q = 9.29 × 10^−110^). Finally, deletion of the SRR124–134 cluster reduced *SOX2* expression back to the levels found in normal breast epithelium (*P* = 0.48, Tukey's test) (85,86) (Figure 2G). Together, these data confirm that the SRR124–134 cluster drives *SOX2* overexpression in BRCA and LUAD.

### SOX2 regulates pathways associated with epithelium development in luminal A BRCA

**Figure 3.**
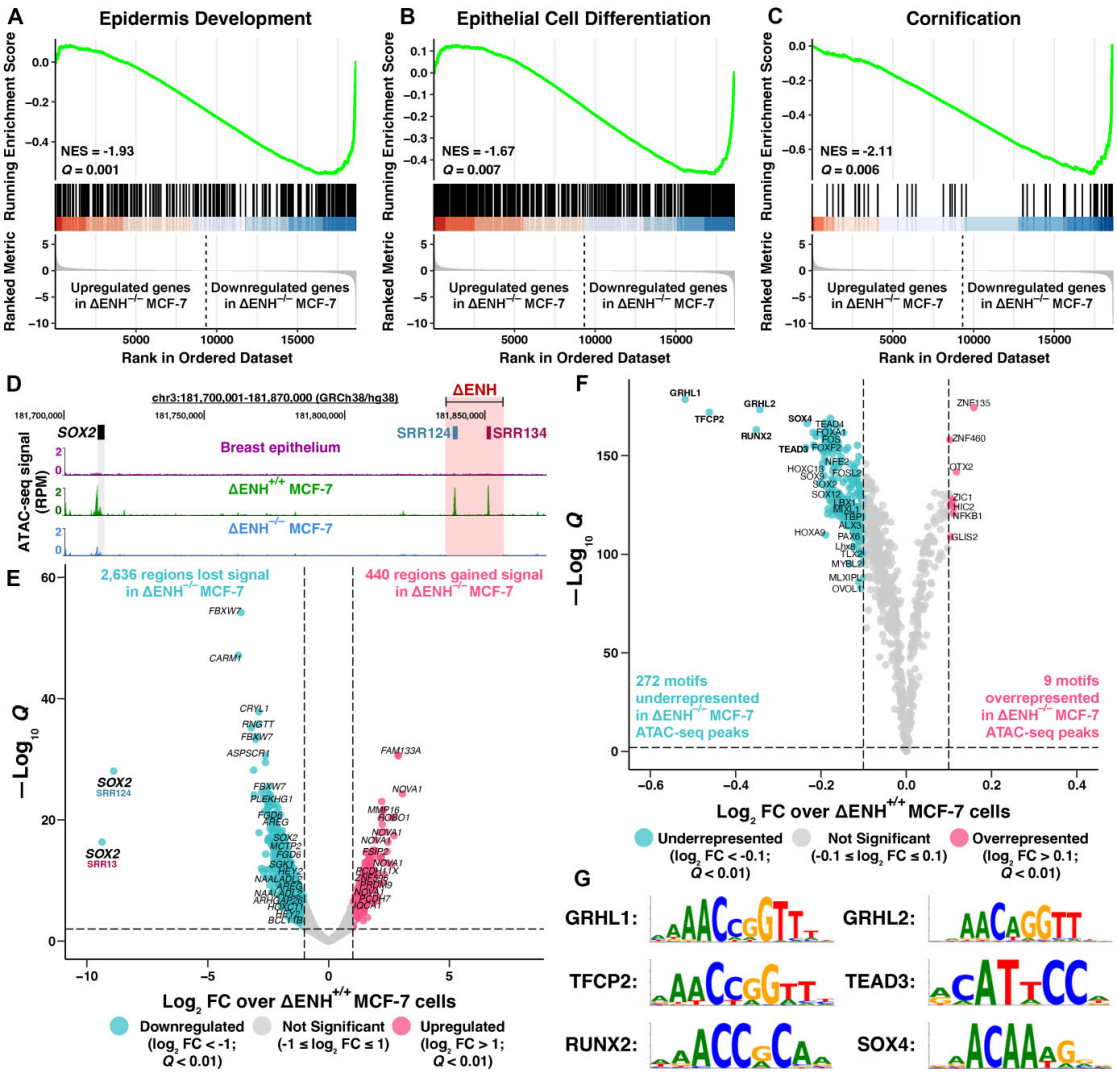
*SOX2* down-regulation impacts chromatin accessibility in luminal A BRCA. (**A–C**) GSEA in the transcriptome of ΔENH^–/–^ compared with ΔENH^+/+^ MCF-7 cells. Genes were ranked according to their change in expression (log_2_ FC). A subset of Gene Ontology (GO) terms significantly enriched among down-regulated genes in ΔENH^–/–^ MCF-7 cells are displayed, indicated by the NES < 1: (**A**) epidermis development, (**B**) epithelial cell differentiation and (**C**) cornification. GSEA was performed using clusterProfiler (94) with an FDR-adjusted *Q* < 0.05 threshold. Green line: running enrichment score. (**D**) UCSC Genome Browser (102) view of the SRR124–134 deletion in ΔENH^–/–^ MCF-7 cells with ATAC-seq tracks from breast epithelium (86), ΔENH^+/+^ and ΔENH^–/–^ MCF-7 cells. (**E**) Volcano plot with differential ATAC-seq analysis between ΔENH^–/–^ and ΔENH^+/+^ MCF-7 cells. Blue: 2638 regions that lost (log_2_ FC < –1; FDR-adjusted *Q* < 0.01) chromatin accessibility in ΔENH^–/–^ MCF-7 cells. Pink: 440 regions that gained (log_2_ FC > 1; *Q* < 0.01) chromatin accessibility in ΔENH^–/–^ MCF-7 cells. Gray: 132 726 regions that retained chromatin accessibility in ΔENH^–/–^ MCF-7 cells (–1 ≤ log_2_ FC ≤ 1). Regions were labeled with their closest gene within a ± 1 Mb distance threshold. Differential chromatin accessibility analysis was performed using diffBind (96). (**F**) Volcano plot with ATAC-seq footprint analysis of differential transcription factor binding in ΔENH^–/–^ compared with ΔENH^+/+^ MCF-7 cells. Blue: 272 under-represented (log_2_ FC < –0.1; FDR-adjusted *Q* < 0.01) motifs in ATAC-seq peaks from ΔENH^–/–^ MCF-7 cells. Pink: nine over-represented (log_2_ FC > 0.1; *Q* < 0.01) motifs in ATAC-seq peaks from ΔENH^–/–^ MCF-7 cells. Gray: 560 motifs with no representative change (–0.1 ≤ log_2_ FC ≤ 0.1) within ATAC-seq peaks from ΔENH^–/–^ MCF-7 cells. (**G**) Sequence motifs of the top six transcription factors with the lowest binding score in ΔENH^–/–^ compared with ΔENH^+/+^ MCF-7 cells: GRHL1, TFCP2, RUNX2, GRHL2, TEAD3 and SOX4. Footprint analysis was performed using TOBIAS (97) utilizing the JASPAR 2022 motif database (79).

Given the established role of SOX2 in regulating proliferation and differentiation pathways in other epithelial cells (40,130), we decided to further investigate the molecular function of SOX2 in luminal A BRCA cells by leveraging our *SOX2*-depleted ΔENH^–/–^ MCF-7 cell model. GSEA showed a significant (FDR-adjusted *Q* < 0.05) depletion of multiple epithelium-associated processes within the transcriptome of ΔENH^–/–^ MCF-7 cells, as indicated by the normalized enrichment score (NES) < 1 (Supplementary Table S20). These processes included epidermis development (NES = –1.93; *Q* = 0.001; Figure 3A), epithelial cell differentiation (NES = –1.67; *Q* = 0.007; Figure 3B) and cornification (NES = –2.11; *Q* = 0.006; Figure 3C). Cornification is the process of terminal differentiation of epidermal cells, wherein these cells undergo a specialized form of programmed cell death to produce a layer of flattened, dead cells with a high keratin content (reviewed in 131). This suggests that SOX2 has a pivotal role in regulating epithelial development and differentiation pathways in luminal A BRCA cells.

SOX2 is a pioneer transcription factor that associates with its motif in heterochromatin (132) and recruits chromatin-modifying complexes (133) in embryonic and reprogrammed stem cells. We performed ATAC-seq in ΔENH^–/–^ MCF-7 cells and compared chromatin accessibility with ΔENH^+/+^ MCF-7 cells to identify genome-wide loci that are dependent on SOX2 to remain accessible in luminal A BRCA. As expected, the ATAC-seq signal from all replicates was highly enriched around the gene TSS (Supplementary Figure S3A), with both ΔENH^+/+^ (Supplementary Figure S3B) and ΔENH^–/–^ (Supplementary Figure S3C) MCF-7 cells having higher chromatin accessibility at the TSS of highly expressed genes. Correlation analysis also confirmed the clustering of all three replicates from each genotype (Supplementary Figure S3D). Including the SRR124–134 cluster and pSOX2 (Figure 3D), a total of 3076 regions of 500 bp had significant (|log_2_ FC| > 1; FDR-adjusted *Q* < 0.01) changes in chromatin accessibility in ΔENH^–/–^ compared with ΔENH^+/+^ MCF-7 cells (Figure 3E; Supplementary Table S21). Most regions (86%, 2636 regions) significantly lost chromatin accessibility in ΔENH^–/–^ MCF-7 cells and 76% (2024 regions) of these regions also gained chromatin accessibility in ΔENH^+/+^ MCF-7 cells compared with normal breast epithelium (86) (Supplementary Table S22). Together, this supports the important role that SOX2 plays in modulating the chromatin accessibility changes acquired in luminal A BRCA.

We used TOBIAS (97) to further analyze changes in transcription factor footprints within differential ATAC-seq peaks between ΔENH^–/–^ and ΔENH^+/+^ MCF-7 cells. From 841 vertebrate motifs (79), we found a total of 281 motifs with a significant (|log_2_ FC| > 0.1; FDR-adjusted *Q* < 0.01) differential binding score (Figure 3F; Supplementary Table S23). Most of these motifs (97%, 272 motifs) were under-represented within ATAC-seq peaks in ΔENH^–/–^ compared with ΔENH^+/+^ MCF-7 cells, indicating that reduced *SOX2* expression affects the binding of multiple other transcription factors. Among them, the GRHL1 (log_2_ FC = –0.519; *Q* = 3 × 10^−179^), TFCP2 (log_2_ FC = –0.462; *Q* = 1.03 × 10^−172^), RUNX2 (log_2_ FC = –0.352; *Q* = 8.02 × 10^−164^), GRHL2 (log_2_ FC = –0.343; *Q* = 4.43 × 10^−174^), TEAD3 (log_2_ FC = –0.235; *Q* = 9.74 × 10^−155^) and SOX4 (log_2_ FC = –0.232; *Q* = 5.33 × 10^−167^) motifs (Figure 3G) had the most reduced binding score in ΔENH^–/–^ MCF-7 cells compared with ΔENH^+/+^ MCF-7 cells. These factors belong to three main JASPAR (79) motif clusters: GRHL/TFCP (cluster 33; aaAACAGGTTtcAgtt), RUNX (cluster 60; ttctTGtGGTTttt), TEAD (cluster 2; tccAcATTCCAggcCTTta) and SOX (cluster 8; acggaACAATGgaagTGTT). The SOX cluster also included the SOX2 (log_2_ FC = –0.175; *Q* = 6.61 × 10^−139^) motif.

Next, we aimed to analyze ChIP-seq data from transcription factors within these motif clusters in MCF-7 cells. We utilized two published datasets: GRHL2 (107) and RUNX2 (134). Regions that lost (log_2_ FC < –1; *Q* < 0.01) chromatin accessibility in ΔENH^–/–^ compared with ΔENH^+/+^ MCF-7 cells significantly (*P* < 2 × 10^−16^, hypergeometric test) overlapped regions with binding of either of these transcription factors. Among the 2636 regions that lost chromatin accessibility, 40% (750 regions) also show GRHL2 binding (Supplementary Figure S3E), whereas 21% (552 regions) share RUNX2 binding (Supplementary Figure S3F). In addition, we found multiple SOX motifs significantly (FDR-adjusted *Q* < 0.001) enriched within peaks from both GRHL2 (Supplementary Table S24) and RUNX2 (Supplementary Table S25) ChIP-seq datasets, further suggesting that SOX2 collaborates with GRHL2 and RUNX2 to maintain chromatin accessibility in luminal A BRCA. Expression levels of either *GRHL2* or *RUNX2*, however, were not significantly affected by *SOX2* down-regulation in ΔENH^–/–^ MCF-7 cells (–1 ≤ log_2_ FC ≤ 1; Supplementary Table S19), indicating that they are not directly regulated by SOX2 at the transcriptional level but may interact at the protein level.

### The SRR124–134 cluster is associated with *SOX2* overexpression in primary tumors

**Figure 4.**
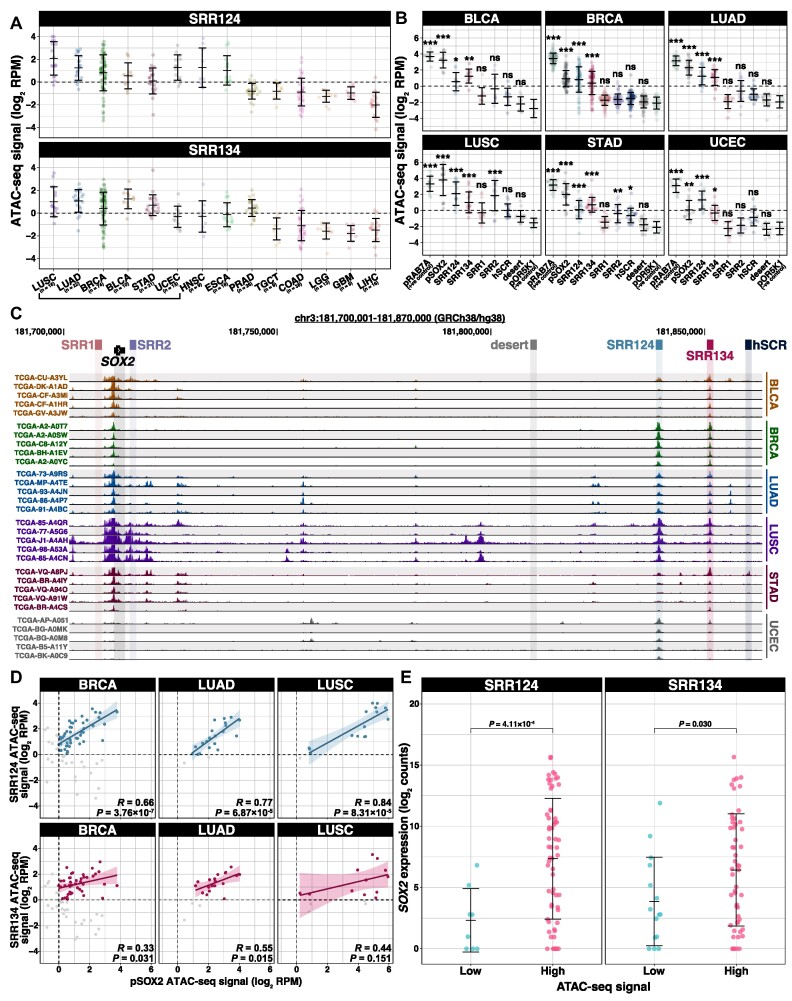
The SRR124–134 cluster is associated with *SOX2* overexpression in cancer patient tumors. (**A**) ATAC-seq signal (log_2_ RPM) at SRR124 and SRR134 for 294 patient tumors from 14 cancer types (100). Cancer types are sorted in descending order by the median signal between all three regions. Dashed line: regions with a sum of reads above our threshold (log_2_ RPM > 0) were considered ‘accessible’. Error bars: SD. Underscore: top six cancer types with the highest ATAC-seq median signal. (**B**) ATAC-seq signal (log_2_ RPM) at the *RAB7A* promoter (pRAB7A), *SOX2* promoter (pSOX2), SRR1, SRR2, SRR124, SRR134, hSCR and a desert region within the *SOX2* locus (desert) compared with the background signal at the repressed *OR5K1* promoter (pOR5K1) in BLCA (*n* = 10), BRCA (*n* = 74), LUAD (*n* = 22), LUSC (*n* = 16), STAD (*n* = 21) and UCEC (*n* = 13) patient tumors. Dashed line: regions with a sum of reads above our threshold (log_2_ RPM > 0) were considered ‘accessible’. Error bars: SD. Significance analysis by Dunn's test with Holm correction (*P < 0.05, **P < 0.01, ***P < 0.001, ns: not significant). (**C**) UCSC Genome Browser (102) visualization of the *SOX2* region with ATAC-seq data from BLCA, BRCA, LUAD, LUSC, STAD and UCEC patient tumors (*n* = 5 in each cancer type) (100). ATAC-seq reads were normalized by library size (RPM). Scale: 0–250 RPM. (**D**) ATAC-seq signal at SRR124 and SRR134 regions against ATAC-seq signal for the *SOX2* promoter (pSOX2) from 74 BRCA, 22 LUAD and 16 LUSC patient tumors. Correlation is shown for accessible chromatin (log_2_ RPM > 0). Gray: tumors with closed chromatin (log_2_ RPM < 0) at either region, not included in the correlation analysis. Significance analysis by Pearson correlation. Bold line: fitted linear regression model. Shaded area: 95% confidence region for the regression fit. (**E**) Comparison of log_2_-normalized *SOX2* transcript levels (log_2_ counts) between BRCA, LUAD and LUSC patient tumors according to the chromatin accessibility at SRR124 and SRR134 regions. Chromatin accessibility at each region was considered ‘low’ if log_2_ RPM < –1, or ‘high’ if log_2_ RPM > 1. RNA-seq reads were normalized to library size using DESeq2 (88). Error bars: SD. Significance analysis by a two-sided *t*-test with Holm correction.

With the confirmation that the SRR124–134 cluster drives *SOX2* overexpression in the BRCA and LUAD cell lines, we investigated chromatin accessibility at this enhancer cluster within primary tumors isolated from cancer patients. By analyzing the pan-cancer ATAC-seq dataset from TCGA (100), we found that SRR124 and SRR134 are most accessible within LUSC, LUAD, BRCA, bladder carcinoma (BLCA), stomach adenocarcinoma (STAD) and uterine endometrial carcinoma (UCEC) patient tumors (Figure 4A). We also quantified the ATAC-seq signal at six other regions: the *SOX2* embryonic-associated enhancers (SRR1, SRR2 and hSCR), the *SOX2* promoter (pSOX2), a gene regulatory desert with no enhancer features located between the *SOX2* gene and the SRR124–134 cluster (desert), and the promoter of the housekeeping gene *RAB7A* (pRAB7A, positive control). We then compared the chromatin accessibility levels at each of these regions with the promoter of the repressed olfactory gene *OR5K1* (pOR5K1, negative control). Both SRR124 and SRR134 showed significantly increased (*P* < 0.05, Holm-adjusted Dunn's test) chromatin accessibility when compared with pOR5K1 in BLCA (SRR124 *P* = 0.014; SRR134 *P* = 1.52 × 10^−3^; Holm-adjusted Dunn's test), BRCA (SRR124 *P* = 1.70 × 10^−20^; SRR134 *P* = 1.03 × 10^−16^), LUAD (SRR124 *P* = 6.76 × 10^−7^; SRR134 *P* = 3.26 × 10^−6^), LUSC (SRR124 *P* = 1.62 × 10^−6^; SRR134 *P* = 7.08 × 10^−4^), STAD (SRR124 *P* = 1.15 × 10^−4^; SRR134 *P* = 1.96 × 10^−7^) and UCEC (SRR124 *P* = 3.15 × 10^−5^; SRR134 *P* = 0.025) patient tumors (Figure 4B).

One potential explanation for increased chromatin accessibility could be locus amplification. While LUSC had high levels of chromatin accessibility probably related to previously described *SOX2* amplifications (58,59,111,112), most patient tumors showed no evidence of locus amplifications extending to the SRR124–134 cluster, as evidenced by the lack of significant (*P* > 0.05) accessibility at the intermediate desert region. In contrast, the SRR124–134 cluster displayed a consistent pattern of accessible chromatin across multiple cancer types: BLCA, BRCA, LUAD, LUSC, STAD and UCEC (Figure 4C). GBM and LGG tumors lacked accessible chromatin at this cluster but displayed increased chromatin accessibility at the SRR1 and SRR2 enhancers (Supplementary Figure S4A; Supplementary Table S26), which is consistent with the evidence that SRR1 and SRR2 drive *SOX2* expression in the neural lineage (23,71,135).

Next, we reasoned that an accessible SRR124–134 cluster drives subsequent *SOX2* transcription within patient tumors. If this was the case, we anticipated finding positive and significantly correlated chromatin accessibility between this enhancer cluster and pSOX2. Indeed, we found that the majority of BRCA (58%), LUAD (82%) and LUSC (69%) tumors have concurrent accessibility (log_2_ RPM > 0) at pSOX2, SRR124 and SRR134. Patient tumors also showed a significant (*P* < 0.05) correlation (Pearson *R*) between accessible chromatin signal at pSOX2 and at both SRR124 and SRR134 in BRCA and LUAD (Figure 4D). LUSC tumors showed a significant correlation between accessible chromatin at pSOX2 and SRR124, but not at SRR134 (Figure 4D). As a negative control, we measured the correlation between chromatin accessibility at pSOX2 and at the *SOX2* desert region and found no significant (*P* > 0.05) correlation in any of these cancer types (Supplementary Figure S4B). We also conducted a similar analysis after segregating BRCA tumors into luminal A, luminal B, HER2^+^ and basal-like subtypes (100,117). Interestingly, we found that both luminal A and luminal B tumors possess a significant (*P* < 0.05) correlation between enhancer accessibility and pSOX2 accessibility, whereas for HER2^+^ tumors the correlation was weaker (Supplementary Figure S4C). Basal-like tumors, on the other hand, display no accessible chromatin at either SRR124 or SRR134. This supports that luminal BRCA and LUAD subtypes are strongly associated with increased accessibility at the SRR124–134 cluster.

Finally, by separating BRCA, LUAD and LUSC patient tumors according to their chromatin accessibility at SRR124 and SRR134, we found that tumors with the most accessible chromatin at each of these regions also significantly (*P* < 0.05, *t*-test) overexpress *SOX2* compared with tumors with low chromatin accessibility at these regions (Figure 4E; Supplementary Table S27). Together, these data are consistent with a model in which increased chromatin accessibility at the SRR124–134 cluster drives *SOX2* overexpression in breast and lung patient tumors.

### *FOXA1* and *NFIB* are upstream regulators of the SRR124–134 cluster

**Figure 5.**
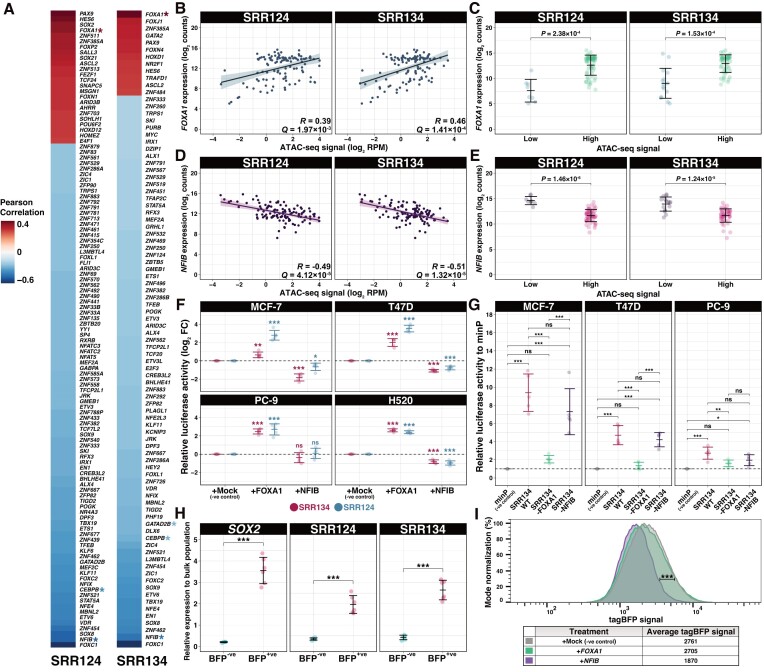
FOXA1 and NFIB are upstream regulators of SRR124 and SRR134. (**A**) Heatmap of the Pearson correlation between transcription factor expression (90) and chromatin accessibility (100) at SRR124 and SRR134 in BRCA, LUAD and LUSC patient tumors (*n* = 111). Transcription factors are ordered according to their correlation to chromatin accessibility at each region. Red: transcription factors with a positive correlation (*R* > 0; FDR-adjusted *Q* < 0.05) to chromatin accessibility. Blue: transcription factors with a negative correlation (*R* < 0; *Q* < 0.05) to chromatin accessibility. Asterisk: transcription factors that show binding at SRR124 or SRR134 by ChIP-seq (85). (**B**) Correlation analysis between *FOXA1* expression (log_2_ counts) and chromatin accessibility (log_2_ RPM) at SRR124 and SRR134 regions in BRCA (*n* = 74), LUAD (*n* = 21) and LUSC (*n* = 16) tumors. RNA-seq reads were normalized to library size using DESeq2 (88). Significance analysis by Pearson correlation (*n* = 111). Bold line: fitted linear regression model. Shaded area: 95% confidence region for the regression fit. (**C**) Comparison of *FOXA1* expression (log_2_ counts) from BRCA, LUAD and LUSC patient tumors according to their chromatin accessibility at the SRR124 and SRR134 regions. Chromatin accessibility at each region was considered ‘low’ if log_2_ RPM < 1, or ‘high’ if log_2_ RPM > 1. RNA-seq reads were normalized to library size using DESeq2 (88). Error bars: SD. Significance analysis by a two-sided *t*-test with Holm correction. (**D**) Correlation analysis between *NFIB* expression (log_2_ counts) and chromatin accessibility (log_2_ RPM) at SRR124 and SRR134 regions in BRCA (*n* = 74), LUAD (*n* = 21) and LUSC (*n* = 16) tumors. RNA-seq reads were normalized to library size using DESeq2 (88). Significance analysis by Pearson correlation (*n* = 111). Boldline: fitted linear regression model. Shaded area: 95% confidence region for the regression fit. (**E**) Comparison of *NFIB* expression (log_2_ counts) from BRCA, LUAD and LUSC patient tumors according to their chromatin accessibility at the SRR124 and SRR134 regions. Chromatin accessibility at each region was considered ‘low’ if log_2_ RPM < 1, or ‘high’ if log_2_ RPM > 1. RNA-seq reads were normalized to library size using DESeq2 (88). Error bars: SD. Significance analysis by a two-sided *t*-test with Holm correction. (**F**) Relative fold change (log_2_ FC) in luciferase activity driven by SRR124 and SRR134 after overexpression of either *FOXA1* or *NFIB* compared with an empty vector (mock negative control, miRFP670). Dashed line: average activity of the mock control. Error bars: SD. Significance analysis by Tukey's test (*n* = 5; *P < 0.05, **P < 0.01, ***P < 0.001, ns: not significant). (**G**) Relative luciferase activity driven by WT, FOXA1-mutated and NFIB-mutated SRR134 constructs compared with a minimal promoter (minP) vector in the MCF-7, PC-9 and T47D cell lines. Dashed line: average activity of minP. Error bars: SD. Significance analysis by Tukey's test (*n* = 5; *P < 0.05, **P < 0.01, ***P < 0.001, ns: not significant). (**H**) RT–qPCR comparison of transcripts at *SOX2*, SRR124 and SRR134 between sorted BFP^−ve^ and BFP^+ve^ MCF-7 cells relative to the unsorted population. Error bars: SD. Significance analysis by paired *t*-test with Holm correction (*n* = 6; ***P < 0.001). (**I**) FACS density plot comparing tagBFP signal between *SOX2*-P2A-tagBFP MCF-7 cells transfected with an empty vector (mock negative control, miRFP670), FOXA1-T2A-miRFP670 or NFIB-T2A-miRFP670. tagBFP signal was acquired from successfully transfected live cells (miRFP^+^/PI^–^) after 5 days post-transfection. Significance analysis by FlowJo's chi-squared T(x) test. T(x) scores >1000 were considered ‘strongly significant’ (***P < 0.001), whereas T(x) scores <100 were considered ‘non-significant’.

Given the evidence that the SRR124–134 cluster is driving *SOX2* overexpression in cancer patient tumors, we investigated which transcription factors regulate this cluster in BRCA, LUAD and LUSC tumors from TCGA (90,100). From a comprehensive list of 1622 human transcription factors (136), we found 115 transcription factors whose expression significantly correlated (FDR-adjusted *Q* < 0.05) with chromatin accessibility at SRR124 and 90 transcription factors whose expression correlated with accessibility at SRR134 (Figure 5A; Supplementary Table S28). From this list, we focused our investigation on FOXA1 and NFIB, which show binding at both SRR124 and SRR134 in ChIP-seq data from MCF-7 cells (85).

The expression of *FOXA1* is positively (Pearson correlation *R* > 0) and significantly correlated to chromatin accessibility at both SRR124 (*R* = 0.39; FDR-adjusted *Q* = 1.97 × 10^−3^) and SRR134 (*R* = 0.46; *Q* = 1.41 × 10^−4^) (Figure 5B). By separating BRCA, LUAD and LUSC patient tumors according to the chromatin accessibility levels at each region, we found that tumors with the most accessible chromatin within SRR124 (*P* = 2.38 × 10^−4^, *t*-test) and SRR134 (*P* = 1.53 × 10^−4^) also significantly overexpress *FOXA1* compared with tumors with low accessibility at these regions (Figure 5C; Supplementary Table S29). On the other hand, we found the expression of *NFIB* to be negatively (*R* < 0) and significantly correlated with chromatin accessibility at both SRR124 (*R* = –0.49; *Q* = 4.12 × 10^−5^) and SRR134 (*R* = –0.51; *Q* = 1.32 × 10^−5^) (Figure 5D). Patient tumors with highly accessible chromatin within SRR124 (*P* = 1.46 × 10^−6^) and SRR134 (*P* = 1.24 × 10^−5^) also display significantly down-regulated *NFIB* expression (Figure 5E; Supplementary Table S30). These data suggest that whereas FOXA1 could be inducing increased accessibility at the SRR124–134 cluster, NFIB expression could counteract FOXA1 by acting as a repressor.

To assess the influence of these transcription factors on enhancer activity, we overexpressed either *FOXA1* or *NFIB* in H520, MCF-7, PC-9 and T47D cells and compared SRR124 and SRR134 enhancer activity measured by luciferase reporter assay with cells transfected with an empty vector (mock). Despite the high endogenous expression of *FOXA1* and *NFIB* in MCF-7 and T47D cells, but not in H520 and PC-9 cells (Supplementary Figure S5A), we found that overexpression of *FOXA1* significantly increased (log_2_ FC > 1; *P* < 0.05, Tukey's test) the enhancer activity of both SRR124 and SRR134 in all four cell lines, whereas *NFIB* overexpression led to a significant decrease (log_2_ FC < 1; *P* < 0.05) in SRR124 and SRR134 enhancer activity in the H520, MCF-7 and T47D cell lines (Figure 5F). This further indicates that *FOXA1* overexpression increases SRR124–134 activity, whereas NFIB represses the enhancer activity of this cluster.

To assess the importance of FOXA1 and NFIB motifs in modulating enhancer activity, we analyzed the SRR134 sequence using the JASPAR2022 motif database (79) and mutated FOXA1 (GTAAACA) or NFIB (TGGCAnnnnGCCAA) motifs to eliminate their binding. We found that mutation of the FOXA1 motif abolished SRR134 enhancer activity measured by luciferase reporter assay compared with the WT SRR134 sequence within MCF-7 (*P* = 1.53 × 10^−5^, Tukey's test), PC-9 (*P* = 1 × 10^−2^) and T47D (*P* = 4.48 × 10^−6^) cells, whereas no significant change (*P* > 0.05) in enhancer activity was found for the NFIB-mutated construct (Figure 5G). These findings underscore the pivotal role of the FOXA1 motif in maintaining SRR134 activity, whereas the NFIB motif is dispensable in this context, consistent with the behavior of a negative regulator when the target activity is elevated.

With the evidence that these two transcription factors are modulating SRR124–134 activity, we investigated their transcriptional effects on *SOX2* expression. We used CRISPR HDR to create an MCF-7 cell line in which the *SOX2* gene is tagged with a 2A self-cleaving peptide (P2A) followed by a blue fluorescent protein (tagBFP). This cell line, MCF-7 *SOX2*-P2A-tagBFP, allows rapid visualization of *SOX2* transcriptional changes by measuring tagBFP signal through FACS. To validate this model, we sorted cells within the top 10% (BFP^+ve^) and bottom 10% (BFP^−ve^) tagBFP signal (Supplementary Figure S5B). We found that BFP^+ve^ cells showed a significant (*P* = 4.25 × 10^−5^, paired *t-*test) increase in *SOX2* expression, and displayed significantly up-regulated transcription of enhancer RNA (eRNA) at SRR124 (*P* = 1.54 × 10^−4^) and SRR134 (*P* = 5.13 × 10^−5^) compared with BFP^−ve^ cells (Figure 5H). This confirms that the tagBFP signal is directly correlated to *SOX2* transcription levels and enhancer output in MCF-7 *SOX2*-P2A-tagBFP cells.

Finally, we overexpressed *FOXA1* or *NFIB* in MCF-7 SOX2-P2A-tagBFP to assess changes in *SOX2* transcription. Although overexpression of *FOXA1* did not significantly [chi-squared T(x) = 63.70] change the tagBFP signal, we found that overexpression of *NFIB* significantly [chi-squared T(x) = 1168.88] reduced the tagBFP signal compared with transfection of an empty vector (mock) (Figure 5I). This confirms the repressive effect of NFIB over *SOX2* expression and illustrates a potential mechanism upstream of *SOX2* that modulates chromatin accessibility at the SRR124–134 cluster and subsequent control of *SOX2* transcription in cancer cells.

### SRR124 and SRR134 are conserved enhancers across mammals and are required for the separation of the anterior foregut

**Figure 6.**
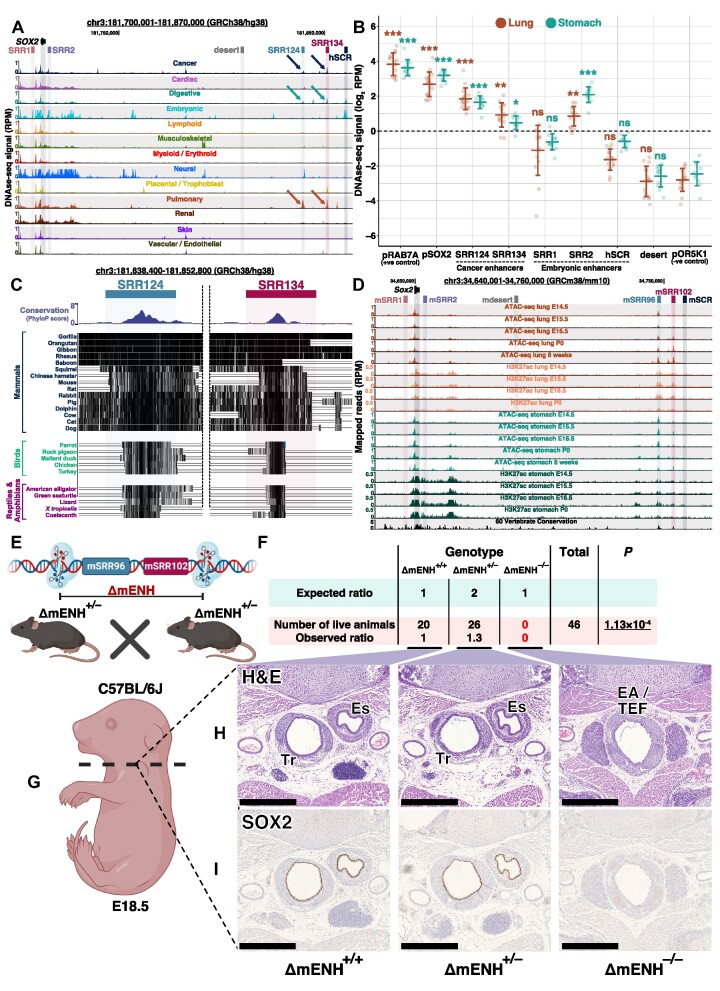
The SRR124 and SRR134 enhancers are conserved across species and are required for the separation of the esophagus and trachea in the mouse. (**A**) UCSC Genome Browser (102) view of the *SOX2* region containing a compilation of chromatin accessibility tracks of multiple human tissues (85,86,137). Arrow: increased chromatin accessibility at the SRR124–134 cluster in cancer and in digestive and respiratory tissues. (**B**) DNase-seq quantification (log_2_ RPM) at the *RAB7A* promoter (pRAB7A), *SOX2* promoter (pSOX2), SRR1, SRR2, SRR124, SRR134, hSCR and a desert region within the *SOX2* locus (desert) compared with the background signal at the repressed *OR5K1* promoter (pOR5K1) in lung and stomach embryonic tissues (85). Dashed line: regions with a sum of reads above our threshold (log_2_ RPM > 0) were considered ‘accessible’. Error bars: SD. Significance analysis by Dunn's test with Holm correction (*P < 0.05, **P < 0.01, ***P < 0.001, ns: not significant). (**C**) UCSC Genome Browser (102) with PhyloP conservation scores (103) at the SRR124 and SRR134 enhancers across mammals, birds, reptiles and amphibians. Black lines: highly conserved sequences. Empty lines: variant sequences. (**D**) UCSC Genome Browser (102) view of the *Sox2* region in the mouse. ATAC-seq and H3K27ac ChIP-seq data from lung and stomach tissues throughout developmental days E14.5 to the eighth post-natal week (85,101). mSRR96: homologous to SRR124. mSRR102: homologous to SRR134. Reads were normalized to library size (RPM). (**E**) Illustration demonstrating the mSRR96–102 enhancer cluster CRISPR deletion (ΔmENH) in C57BL/6J mouse embryos. (**F**) Quantification and genotype of the C57BL/6J progeny from mSRR96–102-deleted crossings (ΔmENH^+/–^). Pups were counted and genotyped at weaning (P21). Significance analysis by chi-squared test to measure the deviation in the number of obtained pups from the expected Mendelian ratio of 1:2:1 (ΔmENH^+/+^:ΔmENH^+/–^:ΔmENH^–/–^). (**G**) Transverse cross-section of fixed E18.5 embryos at the start of the thymus. (**H**) Embryo sections stained with H&E. Scale bar: 500 μm. Es, esophagus; Tr, trachea; EA/TEF, esophageal atresia with distal tracheoesophageal fistula. (**I**) Embryo cross-sections stained for SOX2. Scale bar: 500 μm. Es, esophagus; Tr, trachea; EA/TEF, esophageal atresia with distal tracheoesophageal fistula.

*SOX2* is required for the proper development of multiple tissues (39), including the digestive and respiratory systems in the mouse (25,27,29,31,32,40) and in humans (34–36). Therefore, we questioned whether the SRR124–134 cluster drives *SOX2* expression in additional contexts other than cancer. An analysis of chromatin accessibility data spanning a range of tissue types—cardiac, digestive, embryonic, lymphoid, musculoskeletal, myeloid, neural, placental, pulmonary, renal, skin and vascular tissues (85,86,137)—showed that both SRR124 and SRR134 display increased chromatin accessibility in digestive and respiratory tissues alongside cancer samples (Figure 6A). By comparing DNase-seq signal from fetal lung and stomach tissues (85), we found that both SRR124 (lung *P* = 1.25 × 10^−6^; stomach *P* = 9.64 × 10^−4^; Holm-adjusted Dunn's test) and SRR134 (lung *P* = 1.14 × 10^−3^; stomach *P* = 0.045), together with SRR2 (lung *P* = 1.55 × 10^−3^; stomach *P* = 5.74 × 10^−5^), are significantly more accessible than pOR5K1 (Figure 6B; Supplementary Table S31). This suggests that SRR124 and SRR134 are contributing to *SOX2* expression during the development of the digestive and respiratory systems.

Since critical developmental genes are often controlled by highly conserved enhancers across species (138,139), we hypothesized that the SRR124–134 cluster might regulate *SOX2* expression during the development of other species. By analyzing PhyloP conservation scores (102,103), we discovered that both SRR124 and SRR134 contain a highly conserved core sequence that is preserved across mammals, birds, reptiles and amphibians (Figure 6C). After aligning and comparing enhancer sequences between humans and mice, we found that the core sequences at both SRR124 and SRR134 are highly conserved (> 80%) in the mouse genome (Supplementary Figure S6A). We termed these homologous regions mSRR96 (96 kb downstream of the mouse *Sox2* promoter; homologous to the human SRR124) and mSRR102 (102 kb downstream of the mouse *Sox2* promoter; homologous to the human SRR134). Enhancer feature analysis in the developing lung and stomach tissues in the mouse (85,101) showed that both mSRR96 and mSRR102 display increased chromatin accessibility and H3K27ac signal throughout developmental days E14.5 to the eighth post-natal week (Figure 6D). Interestingly, mSRR96 and mSRR102 display higher ATAC-seq and H3K27ac signal towards the later stages of development in the lungs, but at early stages of development in the stomach. This suggests a distinct spatiotemporal contribution of this homologous cluster to *Sox2* expression during the development of these tissues in the mouse. Furthermore, ATAC-seq quantification showed that both mSRR96 (lung *P* = 5.54 × 10^−5^; stomach *P* = 2.37 × 10^−4^; Holm-adjusted Dunn's test) and mSRR102 (lung *P* = 1.27 × 10^−3^; stomach *P* = 0.046) are significantly more accessible than the repressed promoter of the olfactory gene *Olfr266* (pOlfr266, negative control) during the development of the lungs and stomach in the mouse (Supplementary Figure S6B; Supplementary Table S32). Together, these results suggest a conserved *SOX2* regulatory mechanism across multiple species and support a model in which the SRR124 and SRR134 enhancers and their homologs regulate *SOX2* expression during the development of the digestive and respiratory systems.

To assess the contribution of the mSRR96 and mSRR102 regions to the development of the mouse, we generated a C57BL/6J knockout containing a deletion spanning the mSRR96–102 enhancer cluster (ΔmENH) (Figure 6E). We crossed animals carrying a heterozygous mSRR96–102 deletion (ΔmENH^+/–^) and determined the number of pups alive at weaning (P21) from each genotype. We found a significant (*P* = 1.13 × 10^−4^, Chi-squared test) deviation from the expected Mendelian ratio, with no homozygous mice (ΔmENH^–/–^) alive at weaning (Figure 6F), demonstrating that the mSRR96–102 enhancer cluster is crucial for survival in the mouse. To investigate the resulting phenotype in a homozygous mSRR96–102 enhancer deletion, we collected E18.5 littermate embryos and prepared cross-sections at the thymus level from five animals of each genotype (ΔmENH^+/+^, ΔmENH^+/–^ and ΔmENH^–/–^) (Figure 6G). Similar to other studies that interfered with *Sox2* expression during development (25,32,33), we found that all five ΔmENH^–/–^ embryos developed EA/TEF, where the esophagus and trachea fail to separate during embryonic development (Figure 6H; Supplementary Figure S6C). In contrast, ΔmENH^+/+^ and ΔmENH^+/–^ embryos displayed normal development of the esophageal and tracheal tissues. Immunohistochemistry revealed the complete absence of the SOX2 protein within the EA/TEF tissue in ΔmENH^–/–^ embryos, whereas ΔmENH^+/+^ and ΔmENH^+/–^ embryos showed high levels of SOX2 protein within both the esophagus and tracheal tubes (Figure 6I). Finally, immunofluorescence staining for NKX2.1, a transcription factor associated with the inner epithelium of the respiratory tract (140), showed high protein levels within the inner layer of the EA/TEF tissue in ΔmENH^–/–^ embryos, indicating that this aberrant tissue resembles a tracheal-like structure lacking SOX2 (Supplementary Figure S7A). Together, these results demonstrate that mSRR96 and mSRR102 are required to drive *Sox2* expression during the development and separation of the esophagus and trachea.

## DISCUSSION

Our findings reveal that the SRR124–134 enhancer cluster is essential for *Sox2* expression in the developing digestive and respiratory systems as it is required for the separation of the esophagus and trachea during mouse development. When embryogenesis is complete, *Sox2* expression is down-regulated in most differentiated cell types as its developmental enhancers are decommissioned. We propose that aberrant up-regulation of the pioneer factor *FOXA1* recommissions both SRR124 and SRR134 in tumor cells, driving *SOX2* overexpression in breast and lung adenocarcinoma. Given that SOX2 itself acts as a pioneer transcription factor throughout development, we determined that increased levels of this protein further reprogram the chromatin landscape of cancer cells, binding at multiple regulatory regions, increasing chromatin accessibility, and driving subsequent up-regulation of genes associated with epithelium development. Previous studies have already underscored the indispensable role of SOX2 in both preserving gene expression patterns and orchestrating long-range chromatin interactions in neural stem cells (141), where SOX2 acts as a master regulator (23,142). Considering our observation that the loss of *SOX2* expression leads to a genome-wide reduction in chromatin accessibility and transcription, our results position SOX2 as a central agent in the aberrant activation of gene regulatory pathways that ultimately support a tumor-initiating phenotype in breast and lung adenocarcinomas.

Our discovery that enhancers involved in the development of the digestive and respiratory systems are reprogrammed to support *SOX2* up-regulation during tumorigenesis is in line with previous observations that tumor-initiating cells acquire a less differentiated phenotype (143–146). It is more surprising, however, that the *SOX2* gene is regulated by common enhancers in both breast and lung adenocarcinoma cells as enhancers are usually highly tissue specific (6,138,139,147). Our observation that *FOXA1* expression is significantly correlated to chromatin accessibility at the SRR124–134 cluster and increases the transcriptional output of the SRR124 and SRR134 enhancers provides a mechanistic link between breast and lung developmental programs and cancer progression. FOXA1 is directly involved in the branching morphogenesis of the epithelium in breast (148,149) and lung (150,151) tissues, where SOX2 also plays an important role (27,60). Overexpression of both *FOXA1* (6,9,10,13,152–154) and *SOX2* (55,66,155) have been individually linked to the activation of transcriptional programs associated with multiple types of cancer. Therefore, we propose that FOXA1 is one of the key players responsible for the reprogramming of the SRR124–134 cluster in cancer, which then drives *SOX2* overexpression in breast and lung tumors. It remains intriguing, however, that we were unable to detect a further increase in *SOX2* expression in MCF-7 cells overexpressing *FOXA1* despite observing an up-regulation in SRR124 and SRR134 activity measured by luciferase assay. Since *FOXA1* is already highly expressed in MCF-7 cells, we reason that exogenous overexpression of *FOXA1* may be incapable of further increasing *SOX2* expression if transcriptional levels are already high, such as in the case of MCF-7 cells. Furthermore, our approach to detect changes in *SOX2* transcription using BFP as a fluorescent reporter may have limited our ability to detect small changes in gene expression compared with the higher sensitivity obtained from the luciferase reporter. As mutation of the FOXA1 motif disrupted SRR134 enhancer activity, and this motif is shared among other members of the forkhead box (FOX) transcription factor family (156), it also remains possible that other FOX proteins are involved in activating the SRR124–134 cluster. For example, *FOXM1* overexpression, which also showed binding at both SRR124 and SRR134 in MCF-7 cells, has similarly been associated with poor patient outcomes in multiple types of cancer (157).

In addition to the activating role of FOXA1, we identified NFIB as a negative regulator of *SOX2* expression through inhibition of SRR124–134 activity. NFIB is normally required for the development of multiple tissues (reviewed in 158), including the brain and lungs (159–161), tissues in which *SOX2* expression is also tightly regulated (27,142). In the lungs, NFIB is essential for promoting the maturation and differentiation of progenitor cells (159,160). This is in stark contrast to SOX2, which inhibits the differentiation of lung cells (27). Interestingly, NFIB seems to have paradoxical roles in cancer, acting both as a tumor suppressor and as an oncogene in different tissues (162). Among its tumor suppressor activities, NFIB acts as a barrier to skin carcinoma progression (163), and its down-regulation is associated with dedifferentiation and aggressiveness in LUAD (164). On the other hand, SOX2 promotes skin (66) and lung (165) cancer progression. As an oncogene, *NFIB* promotes cell proliferation and metastasis in STAD (166), where *SOX2* down-regulation is associated with poor patient outcomes (167–169). With this contrasting relationship between *SOX2* and *NFIB* across multiple tissues, we propose that NFIB normally acts as a suppressor of SRR124–134 activity and *SOX2* expression during the differentiation of progenitor cells; down-regulation of *NFIB* expression then results in *SOX2* overexpression during breast and lung tumorigenesis.

We initially hypothesized that SRR1 and SRR2 (70,71,170), and/or the SCR (72,73), might be recommissioned during cancer progression, as stem cell-related enhancers have been shown to acquire enhancer features in tumorigenic cells (171). Although other studies have also proposed the activation of either SRR1 (42,69) or SRR2 (172,173) as the main drivers of *SOX2* overexpression in BRCA, we found no evidence of this mechanism and instead identified the SRR124–134 cluster as the main driver of *SOX2* expression in BRCA and LUAD. Our patient tumor analysis did show that GBM and LGG were the only cancer types that display a unique and consistent pattern of accessible chromatin at SRR1 and SRR2, which is probably related to glioma cells assuming a neural stem cell-like identity to sustain high levels of cell proliferation in the brain (62). In fact, SRR2 deletion was shown to down-regulate *SOX2* and reduce cell proliferation in GBM cells (174), highlighting enhancer specificity to different tumor types. In line with these findings, our observation that PC-9 LUAD cells are dependent on SRR124–134 for *SOX2* transcription, whereas in H520 LUSC cells SRR124–134 is dispensable, again underscores these tumor type-specific regulatory mechanisms. LUSC tumors frequently amplify the *SOX2* locus (58,59,111,112), whereas LUAD tumors do not (175), indicating that different mechanisms are involved in genome dysregulation in these two subtypes of lung cancer. Indeed, we found *FOXA1* expression to be the lowest in H520 cells, which may explain the diminished transcriptional activity of the SRR124–134 cluster in this cell line. Interestingly, a further downstream enhancer cluster located ∼55 kb away from SRR124–134 exhibits high H3K27ac signal and is co-amplified with *SOX2* in H520 cells and other LUSC cell lines (112), revealing an alternative mechanism that could sustain *SOX2* overexpression in the absence of the SRR124–134 cluster in certain types of LUSC but not in LUAD.

Enhancer clusters often contain individual enhancers with partially redundant functions (128,176,177). Our analyses positioned SRR134 as the most potent enhancer within the SRR124–134 cluster. This is not surprising since SRR134 also shows a higher amount of transcription factor binding in MCF-7 cells, a key feature associated with enhancer activity (123). However, while both SRR124 and SRR134 display similar chromatin accessibility in MCF-7 cells, PC-9 cells showed much greater accessibility at the SRR134 enhancer, whereas T47D and H520 cells showed a more accessible SRR124 region. Given that *SOX2* expression is more elevated in MCF-7, T47D and H520 compared with PC-9 cells, we postulate that simultaneous activation of both SRR124 and SRR134 enhancers may be crucial for optimal *SOX2* transcription. Another distinguishing feature between these enhancers is the exclusive binding of CTCF at SRR124. CTCF is a transcription factor involved in chromatin structure and distal enhancer–promoter loop formation at some loci (178,179). Based on these findings, we propose that SRR124 acts as a tether between pSOX2 and SRR134, the latter functioning as a docking region for the binding of multiple transcription factors that ultimately drive *SOX2* overexpression. Therefore, in a scenario where both enhancers are accessible, we believe the chromatin dynamics facilitate enhanced interactions between pSOX2 and the entire SRR124–134 cluster, ultimately elevating the transcription of *SOX2*.

Deletion of mSRR96–102, a homolog of the human SRR124–134 cluster, resulted in EA/TEF, which is also observed in human cases with *SOX2* heterozygous mutations (34–36). A recent study showed that insertion of a CTCF insulation cluster downstream of the *Sox2* gene, but upstream of mSRR96–102, disrupts *Sox2* expression, impairs separation of the esophagus and trachea, and results in perinatal lethality due to EA/TEF in the mouse (33). This was of particular interest for understanding enhancer functional nuances during development since the SCR, which is required for *Sox2* transcription at implantation, can partially overcome the insulator effect of this insertion. The authors proposed that enhancer density might explain the EA/TEF phenotype, as chromatin features suggested that enhancers in the developing lung and stomach tissues might be spread over a 400 kb domain (33). However, the 6 kb deletion that removes the mSRR96–102 cluster causing EA/TEF suggests that this is not the case. Instead, we propose that the sensitivity of each cell type to gene dosage is behind the differing ability of CTCF to block distal enhancers. This is based on two observations: in humans, heterozygous *SOX2* mutations are linked with the anophthalmia–esophageal–genital syndrome (34–36); in mice, hypomorphic *Sox2* alleles display similar phenotypes in the eye (24) and EA/TEF (25,32). This suggests that cells from the peri-implantation phase are less sensitive to lower *Sox2* dosages compared with cells from the developing airways and digestive systems in both species, and explains the aberrant phenotypes observed at term.

Overall, our findings illustrate how *cis-*regulatory regions can similarly drive gene expression in both normal and diseased contexts and serve as a prime example of how decommissioned developmental enhancers may be reprogrammed during tumorigenesis. The fact that we have found a digestive/respiratory-associated enhancer cluster driving gene expression in a non-native context such as BRCA remains intriguing and reinforces a model in which tumorigenic cells often revert to a progenitor-like state that combines *cis-*regulatory features of progenitor cells from multiple developing lineages (6). This ‘dys-differentiation’ mechanism seems to be centered around the overexpression of a few key development-associated pioneer transcription factors such as FOXA1 and SOX2. Identifying additional mechanisms that regulate the reprogramming of these enhancers could lead to new approaches to target tumor-initiating cells that depend on *SOX2* overexpression.

## Supplementary Material

gkad734_Supplemental_files

## Data Availability

Sequencing and processed data files were submitted to the Gene Expression Omnibus (GEO; https://www.ncbi.nlm.nih.gov/geo/) repository (GSE132344).

## Notes

Present address: Jennifer A. Mitchel, Department of Cell and Systems Biology, University of Toronto, Toronto, Canada.
