# Supplementary material for: Epigenetic reprogramming of a distal developmental enhancer cluster drives SOX2 overexpression in breast and lung adenocarcinoma
Source: Nucleic Acids Res. 2023 Sep 22;51(19):10109–31. doi: 10.1093/nar/gkad734 (PMC10602899; doi:10.1093/nar/gkad734)
Supplement: gkad734_Supplemental_files [file gkad734_supplemental_files.zip › SUPPORTING INFORMATION Abatti et al NAR Aug29.docx]

**SUPPORTING INFORMATION for****:**

**Epigenetic reprogramming of a distal developmental enhancer cluster drives *SOX2* overexpression in breast and lung adenocarcinoma**

Author List and Affiliations:

Luis E. Abatti^1^, Patricia Lado-Fernández^2,3^, Linh Huynh^4^, Manuel Collado^2^, Michael M. Hoffman^4,5,6,7^, and Jennifer A. Mitchell^1,8^

^1^Department of Cell and Systems Biology, University of Toronto, Toronto, Ontario, Canada

^2^Laboratory of Cell Senescence, Cancer and Aging, Health Research Institute of Santiago de Compostela (IDIS), Xerencia de Xestión Integrada de Santiago (XXIS/SERGAS), Santiago de Compostela, Spain

^3^Department of Physiology and Center for Research in Molecular Medicine and Chronic Diseases (CiMUS), Universidade de Santiago de Compostela, Santiago de Compostela, Spain

^4^Princess Margaret Cancer Centre, University Health Network, Toronto, Ontario, Canada

^5^Department of Medical Biophysics, University of Toronto, Toronto, Ontario, Canada

^6^Department of Computer Science, University of Toronto, Toronto, Ontario, Canada

^7^Vector Institute for Artificial Intelligence, Toronto, Ontario, Canada

^8^Laboratory Medicine and Pathobiology, University of Toronto, Toronto, Ontario, Canada

Corresponding authors:

ja.mitchell@utoronto.ca, luis.abatti@mail.utoronto.ca

Current address:

Department of Cell and Systems Biology, University of Toronto, Toronto, Canada

**
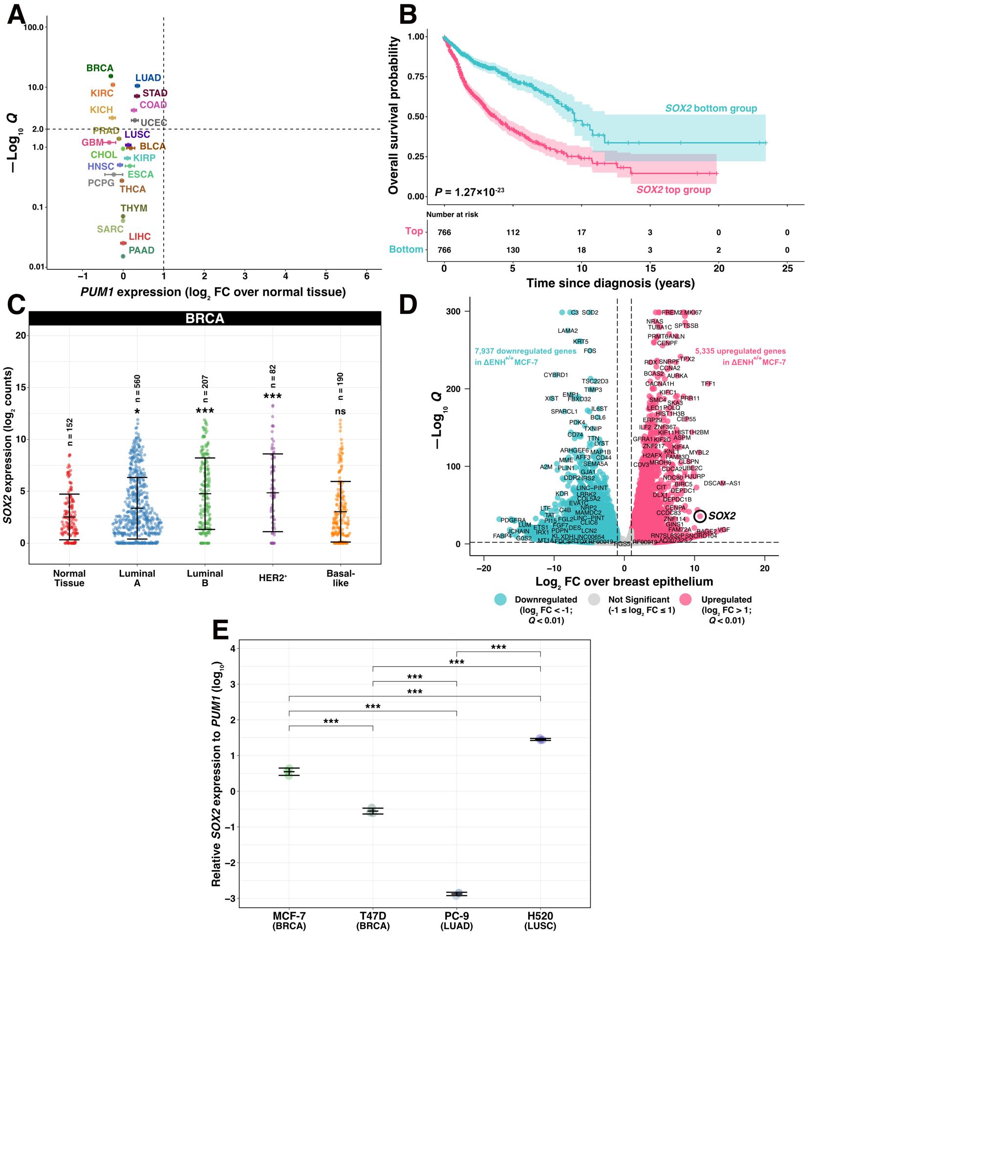
**

**Supplementary Figure S1: (A)** Super-logarithmic volcano plot of *PUM1* expression from RNA-seq of 21 cancer types compared to normal tissue. Cancer types with log_2_ FC > 1 and FDR-adjusted *Q* < 0.01 were considered to significantly overexpress *PUM1*. Error bars: standard deviation. **(B)** Kaplan-Meier plot of overall survival against time since diagnosis for 3,064 patients with BRCA (n = 1089), COAD (n = 453), GBM (n = 153), LIHC (n = 370), LUAD (n = 504), and LUSC (n = 495) tumors. We divided patients into four equal groups and compared two groups: high *SOX2* expression (range: 10.06–16.36 log_2_ counts) and low *SOX2* expression (range: 0–1.67 log_2_ counts). RNA-seq reads were normalized to library size using DESeq2. Significance analysis by logrank test. The shadowed area represents the 95% confidence interval. **(C)** Comparison of *SOX2* expression (log_2_ counts) between luminal A (n = 560), luminal B (n = 207), HER2+ (n = 82), basal-like (n = 190) breast cancer subtypes and normal mammary tissue (n = 152). RNA-seq reads were normalized to library size using DESeq2. Error bars: standard deviation. Significance analysis by Tukey’s test (*** *P* < 0.001, * *P* < 0.05, ns: not significant). **(D)** Volcano plot with DESeq2 differential expression analysis between ΔENH^+/+^ MCF-7 cells and breast epithelium. Blue: 7,937 genes that significantly lost expression (log_2_ FC < -1; FDR-adjusted *Q* < 0.01) in ΔENH^+/+^ MCF-7 cells. Pink: 5,335 genes that significantly gained expression (log_2_ FC > 1; *Q* < 0.01) in ΔENH^+/+^ MCF-7 cells. Grey: 25,342 genes that maintained similar (-1 ≤ log_2_ FC ≤ 1) expression between ΔENH^+/+^ MCF-7 and breast epithelium cells. **(E)** RT-qPCR analysis of *SOX2* transcript levels in the H520, MCF-7, PC-9, and T47D cell lines. Error bars: standard deviation. Significance analysis by Tukey’s test (n = 3; *** *P* < 0.001).

**
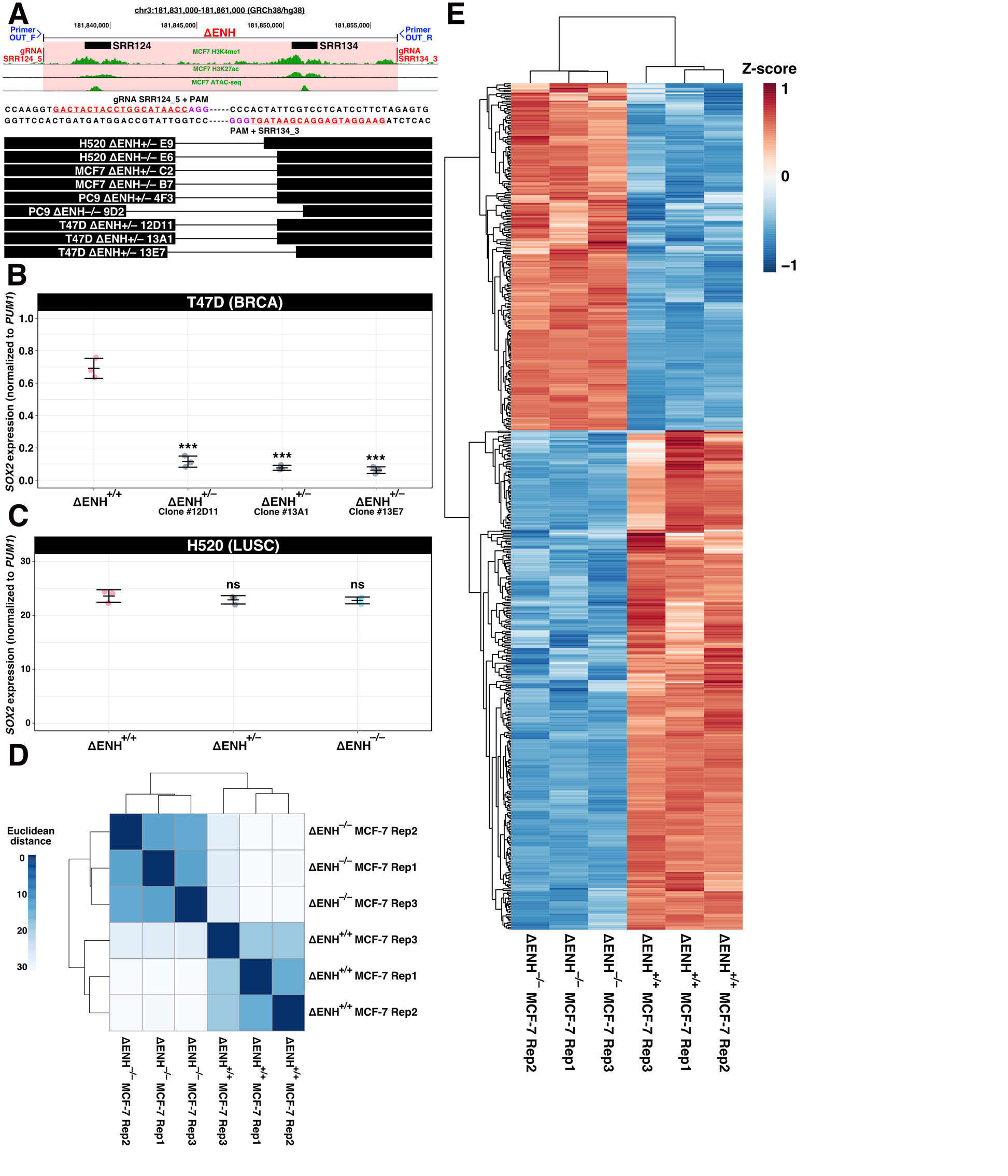
**

**Supplementary Figure S2:** **(A)** UCSC Genome Browser view of the SRR124–134 deletion. Genomic sequences from ΔENH clones were obtained by Sanger sequencing. Red: gRNA sequences. Blue: primers used to amplify the deleted region. Purple: PAM sequences. Green: H3K4me1 and H3K27ac ChIP-seq and ATAC-seq data from MCF-7 cells. White: cell line and clone number. Thick bars: genomic sequence is present. Thin lines: missing sequences from deleted clone. **(B)** RT-qPCR analysis of *SOX2* transcript levels in three independent SRR124–134 heterozygous-deleted (ΔENH^+/–^) T47D clonal isolates compared to WT (ΔENH^+/+^) cells. Error bars: standard deviation. Significance analysis by Dunnett's test (n = 3; *** *P* < 0.001). **(C)** RT-qPCR analysis of *SOX2* transcript levels in SRR124–134 heterozygous- (ΔENH^+/–^) and homozygous- (ΔENH^–/–^) deleted H520 (LUSC) cells compared to WT (ΔENH^+/+^) cells. Error bars: standard deviation. Significance analysis by Dunnett's test (n = 3, ns: not significant). **(D)** Euclidean distance pairwise comparison between ΔENH^+/+^ and ΔENH^–/–^ MCF-7 replicates (n = 3) using variance stabilizing transformed RNA-seq reads from DESeq2. Darker colors indicate a higher correlation. **(E)** Euclidean hierarchical clustering of 529 differentially expressed genes (|log_2_ FC| > 1; FDR-adjusted *Q* < 0.01) based on RNA-seq analysis between ΔENH^+/+^ and ΔENH^–/–^ MCF-7 replicates (n = 3). Reads were normalized for each gene across treatments (Z-score). Blue color indicates downregulated genes (Z-score < 0). Red color indicates upregulated genes (Z-score > 0).

**
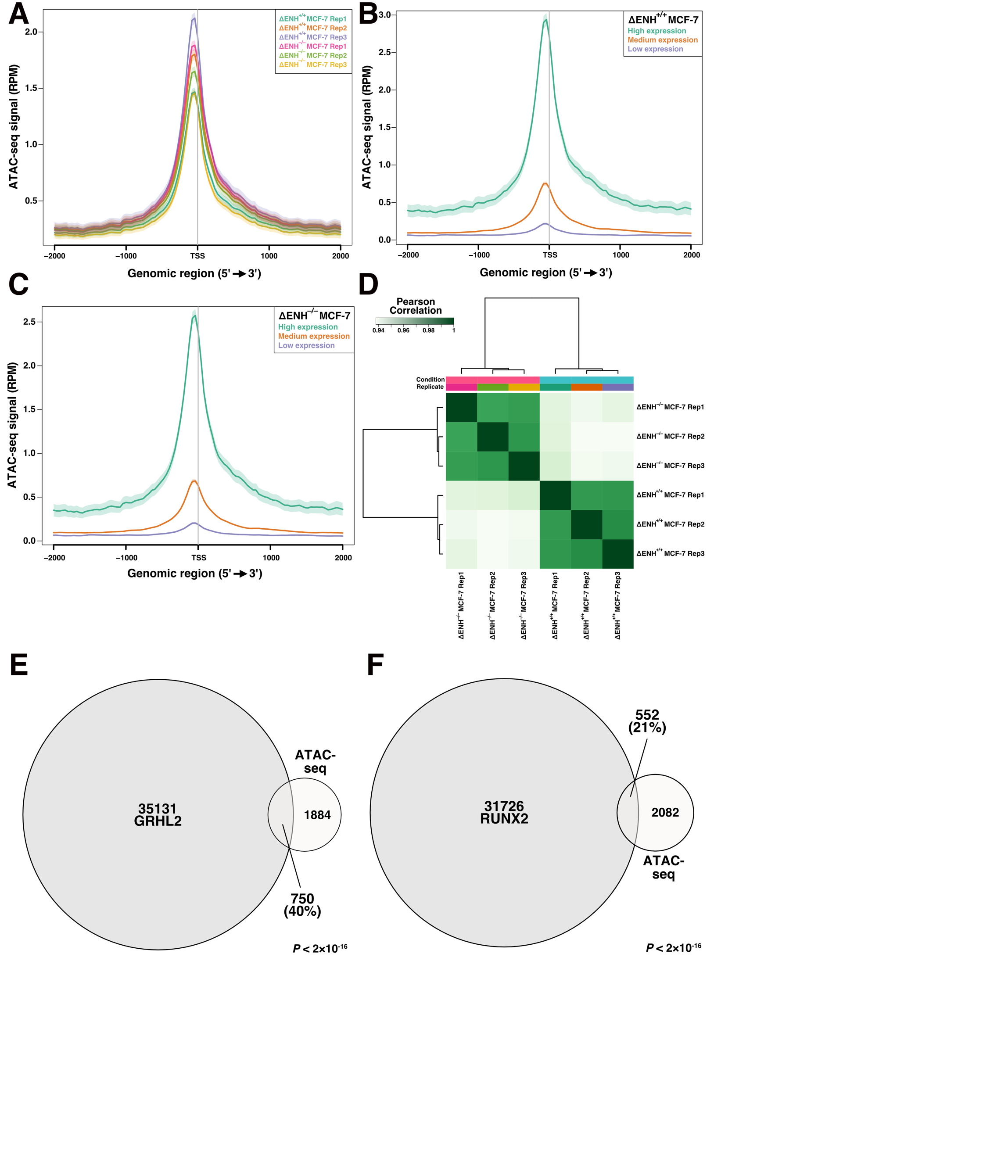
**

**Supplementary Figure S3: (A)** ATAC-seq metagene enrichment plot ± 2 kb around the transcription start site (TSS) across all genes from ΔENH^+/+^ and ΔENH^–/–^ MCF-7 cells (n = 3). Reads were normalized by library size (RPM). Grey: TSS. Shaded area: standard deviation. **(****B)** ATAC-seq metagene enrichment plot ± 2 kb around the transcription start site (TSS) across 12,167 high (average log_2_ counts = 9.12), 12,167 medium (average log_2_ counts = 2.94), and 12,167 low (average log_2_ counts = 0.43) expressed genes in ΔENH^+/+^ MCF-7 cells. Genes were split into each group according to RNA-seq data. RNA-seq reads were normalized to library size using DESeq2. Grey: TSS. (**C)** ATAC-seq metagene enrichment plot ± 2 kb around the transcription start site (TSS) across 12,167 high (average log_2_ counts = 9.10), 12,167 medium (average log_2_ counts = 2.97), and 12,167 low (average log_2_ counts = 0.47) expressed genes in ΔENH^–/–^ MCF-7 cells. Genes were split into each group according to RNA-seq data. RNA-seq reads were normalized to library size using DESeq2. Grey: TSS. **(D)** Pairwise Pearson correlation comparison between ΔENH^+/+^ and ΔENH^–/–^ MCF-7 replicates (n = 3) using ATAC-seq normalized signal from diffBind. Darker colors indicate a higher correlation. **(E)** Overlap between GRHL2 ChIP-seq peaks and ATAC-seq peaks that significantly (log_2_ FC < -1; *P* < 0.01) lost chromatin accessibility in ΔENH^–/–^ compared to ΔENH^+/+^ MCF-7 cells. Significance analysis by the hypergeometric test. **(F)** Overlap between RUNX2 ChIP-seq peaks and ATAC-seq peaks that significantly (log_2_ FC < -1; *P* < 0.01) lost chromatin accessibility in ΔENH^–/–^ compared to ΔENH^+/+^ MCF-7 cells. Significance analysis by the hypergeometric test.

**
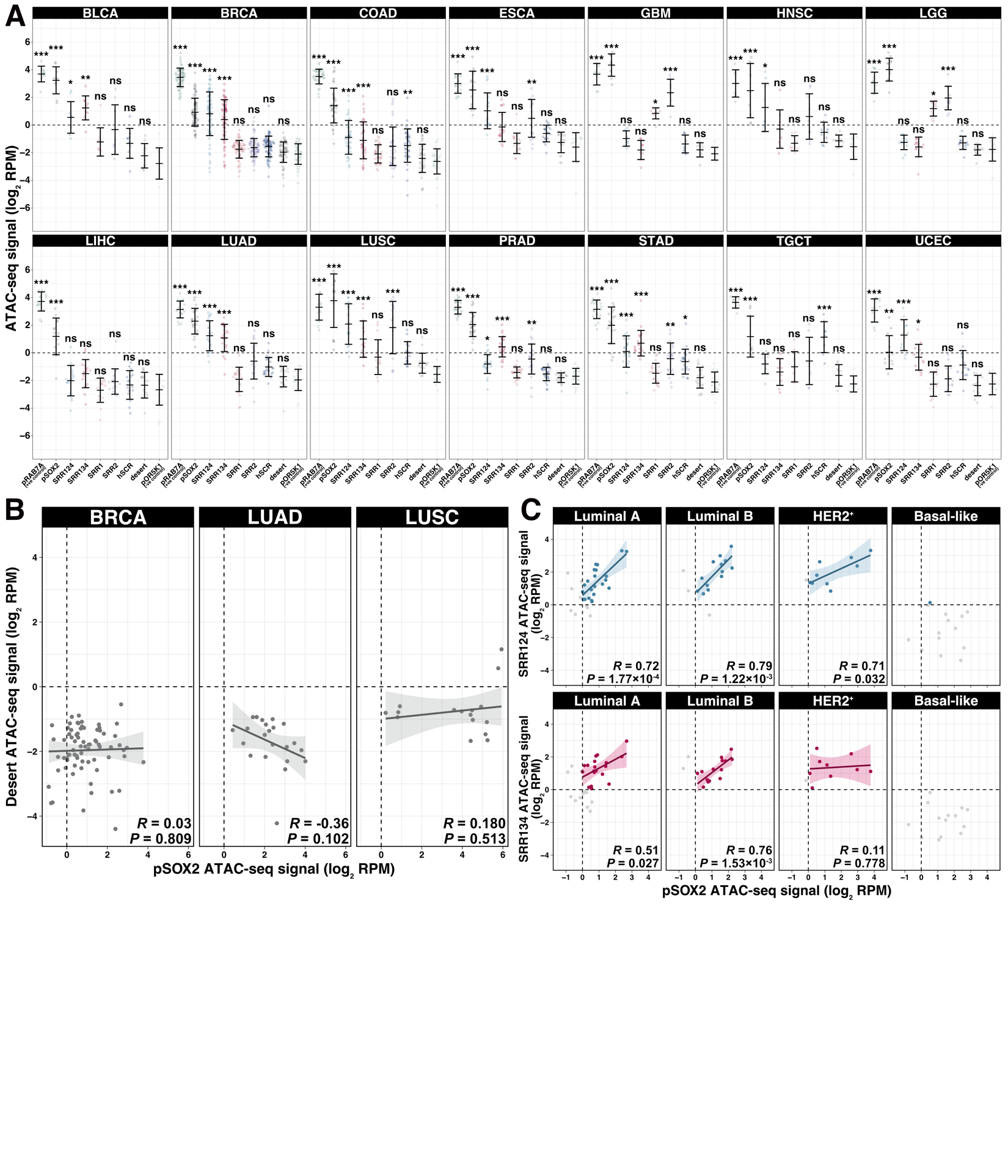
**

**Supplementary Figure S4: (A)** ATAC-seq signal at the *RAB7A* promoter (pRAB7A), *SOX2* promoter (pSOX2), SRR1, SRR2, SRR124, SRR134, human SCR (hSCR) and desert region versus the background signal at the repressed *OR5K1* promoter (pOR5K1) in BLCA (n = 10), BRCA (n = 74), COAD (n = 38), ESCA (n = 18), GBM (n = 9), HNSC (n = 9), LGG (n = 13), LIHC (n = 16), LUAD (n = 22), LUSC (n = 16), PRAD (n = 26), STAD (n = 21), TGCT (n = 9), and UCEC (n = 13) patient tumors. Dashed line: regions with log_2_ RPM > 0 were considered “accessible”. Error bars: standard deviation. Significance analysis by Dunn’s test with Holm correction (* *P* < 0.05, ** *P* < 0.01, *** *P* < 0.001, ns: not significant). **(B)** ATAC-seq signal at the *SOX2* desert region (desert) against ATAC-seq signal for the *SOX2* promoter (pSOX2) from 74 BRCA, 22 LUAD, and 16 LUSC patient tumors. Dashed line: regions with log_2_ RPM > 0 were considered “accessible”. Significance analysis by Pearson correlation. Bolded line: fitted linear regression model. Shaded area: 95% confidence region for the regression fit. **(C)** ATAC-seq signal at SRR124 and SRR134 regions against ATAC-seq signal for the *SOX2* promoter (pSOX2) from BRCA patient tumors separated into luminal A (n = 31), luminal B (n = 16), HER2^+^ (n = 10), and basal-like (n = 14) subtypes. Correlation is shown for accessible chromatin (log_2_ RPM > 0). Grey: tumors with closed chromatin (log_2_ RPM < 0) at either region, not included in the correlation analysis. Significance analysis by Pearson correlation. Bolded line: fitted linear regression model. Shaded area: 95% confidence region for the regression fit.

**
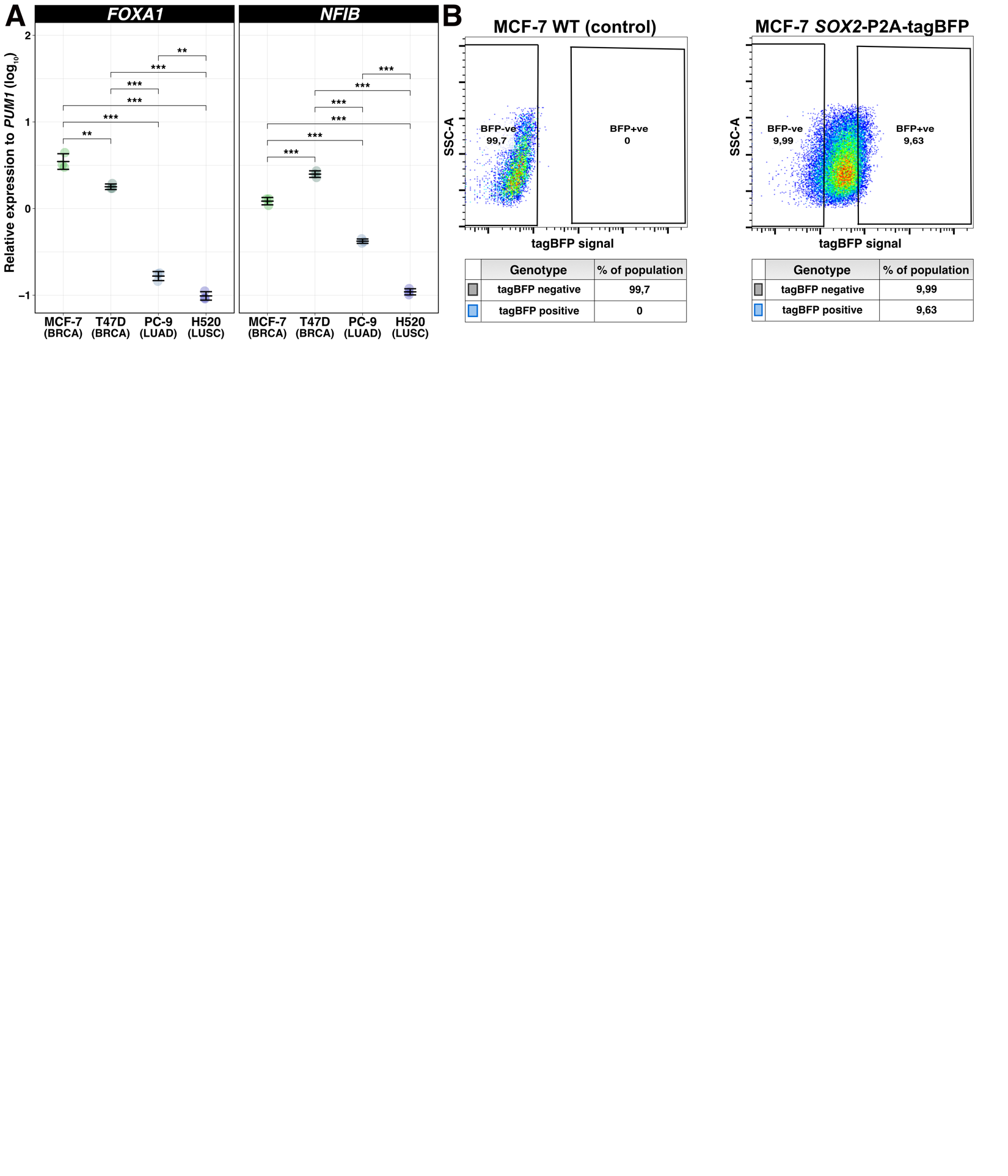
**

**Supplementary Figure S5: (A)** RT-qPCR analysis of *FOXA1 and NFIB* expression in the H520, MCF-7, PC-9, and T47D cell lines. Error bars: standard deviation. Significance analysis by Tukey’s test (n = 3; ** *P* < 0.01, *** *P* < 0.001). **(B)** FACS plot of tagBFP signal (450 nm) over side scatter (SSC) in WT and *SOX2*-P2A-tagBFP MCF-7 cells. Cell populations within the top 10% tagBFP signal were considered “tagBFP positive” (BFP^+ve^), whereas populations within the bottom 10% BFP signal were considered “tagBFP negative” (BFP^-ve^).

**
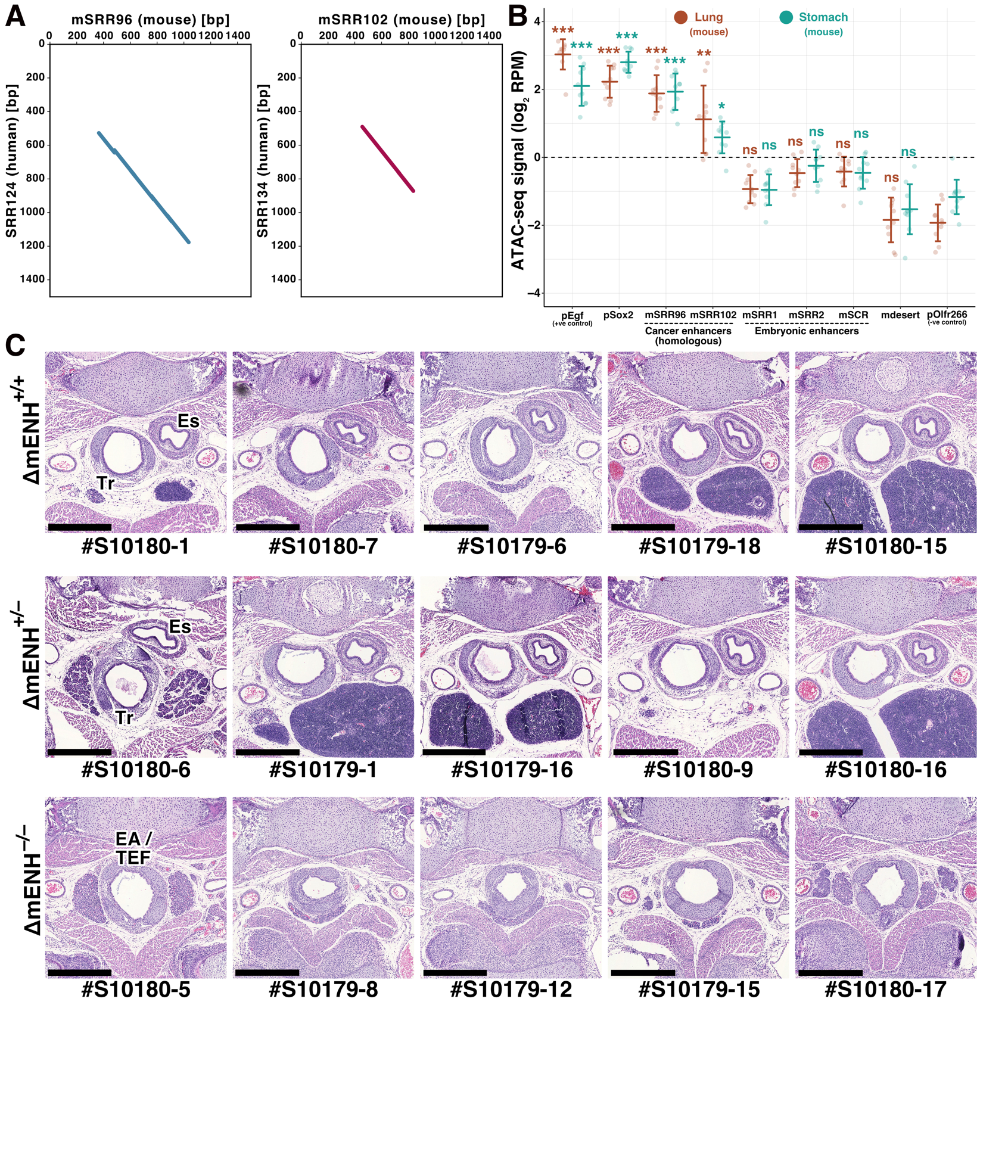
**

**Supplementary Figure S6: (A)** Dot-plot alignment of human (GRCh38/hg38, y-axis) SRR124 and SRR134, and mouse (GRCm38/mm10, x-axis) mSRR96 and mSRR102 homologous sequences (1500 bp). Lines indicate high conservation scores (> 80%) across both species. Sequence alignment using Clustal Omega. **(B)** ATAC-seq quantification (log_2_ RPM) at the promoter of the housekeeping gene *Egf* (pEgf, positive control), *Sox2* promoter (pSox2), mSRR1, mSRR2, mSRR96, mSRR102, mSCR, and a mouse desert (mdesert) region compared to the background signal at the repressed *Olfr266* promoter (pOlfr266) in lung and stomach embryonic tissues from the mouse. mSRR96: homologous to SRR124. mSRR102: homologous to SRR134. Dashed line: regions with a sum of reads above our threshold (log_2_ RPM > 0) were considered “accessible”. Error bars: standard deviation. Significance analysis by Dunn’s test with Holm correction (* *P* < 0.05, ** *P* < 0.01, *** *P* < 0.001, ns: not significant). **(C)** Panel of all E18.5 embryo sections stained with Hematoxylin and Eosin (H&E) (n = 5 for each genotype). Sections were prepared at the thymus level. Scale bar: 500µm. Es: esophagus; Tr: trachea; EA/TEF: esophageal atresia with distal tracheoesophageal fistula.

**
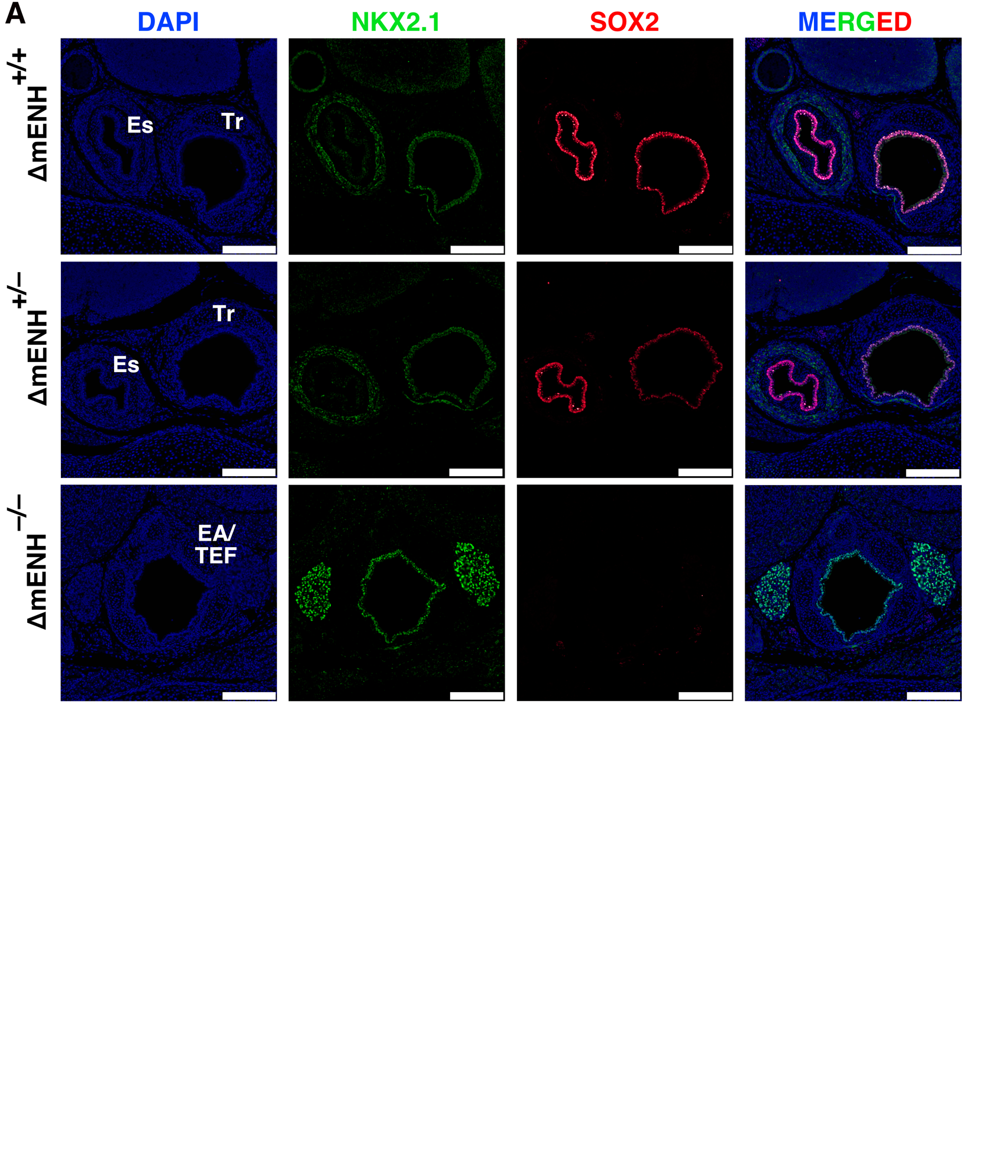
**

**Supplementary Figure S7: (A)** Immunofluorescence staining of ΔmENH^+/+^, ΔmENH^+/–^, and ΔmENH^–/–^ E18.5 embryos with DAPI (blue), NKX2.1 (green), and SOX2 (red). Cross-sections were prepared at the thymus levels. Scale bar: 200µm. Es: esophagus; Tr: trachea; EA/TEF: esophageal atresia with distal tracheoesophageal fistula.

**Supplementary Table S1:** List of gRNA sequences used for CRISPR/Cas9.

**Supplementary Table S2:** DNA sequence surrounding each CRISPR/Cas9 modification.

**Supplementary Table S3:** List of primers used for enhancer cloning.

**Supplementary Table S4:** WT, FOXA1, and NFIB mutated SRR134 sequences.

**Supplementary Table S5:** List of primers used in RT-qPCR experiments.

**Supplementary Table S6:** ENCODE datasets used in this paper.

**Supplementary Table S7:** Coordinates of regions used in genome-wide analysis in humans (GRCh38/hg38).

**Supplementary Table S8:** Coordinates of regions used in genome-wide analysis in the mouse (GRCm38/mm10).

**Supplementary Table S9:** GEO datasets used in this paper.

**Supplementary Table S10:** List of E18.5 mouse embryos utilized in this study.

**Supplementary Table S11:** List of TCGA tumor type abbreviations.

**Supplementary Table S12:** *SOX2* differential expression analysis between primary tumor vs. normal tissue across TCGA cancer types.

**Supplementary Table S13:** *PUM1* differential expression analysis between primary tumor vs. normal tissue across TCGA cancer types.

**Supplementary Table S14:** TCGA cancer patient overall survival analysis relative to *SOX2* expression levels.

**Supplementary Table S15:** TCGA copy number variation (CNV) and *SOX2* expression analysis.

**Supplementary Table S16:** RNA-seq differential expression analysis between ΔENH^+/+^ MCF-7 vs. breast epithelium (ENCODE).

**Supplementary Table S17:** Differential ATAC-seq, H3K4me1, and H3K27ac analysis within ± 1 Mb of the *SOX2* gene in ΔENH^+/+^ MCF-7 vs. Breast epithelium (ENCODE).

**Supplementary Table S18:** ChIA-PET interactions with SRR124 or SRR134 regions in MCF-7 cells.

**Supplementary Table S19:** RNA-seq differential expression analysis comparing ΔENH^–/–^ versus ΔENH^+/+^ MCF-7 cells.

**Supplementary Table S20:** Gene set enrichment analysis (GSEA) in ΔENH^–/–^ versus ΔENH^+/+^ MCF-7 cells.

**Supplementary Table S21:** Significantly changing ATAC-seq peaks in ΔENH^–/–^ versus ΔENH^+/+^ MCF-7 cells.

**Supplementary Table S22:** ATAC-seq peaks that commonly gained signal in ΔENH^+/+^ MCF-7 vs. breast epithelium and lost signal in ΔENH^–/–^ MCF-7 cells.

**Supplementary Table S23:** ATAC-seq footprint analysis in ΔENH^–/–^ vs. ΔENH^+/+^ MCF-7 cells.

**Supplementary Table S24:** ChIP-seq motif analysis of GRHL2 peaks in ΔENH^+/+^ MCF-7 cells.

**Supplementary Table S25:** ChIP-seq motif analysis of RUNX2 peaks in ΔENH^+/+^ MCF-7 cells.

**Supplementary Table S26:** Chromatin accessibility analysis across TCGA cancer types.

**Supplementary Table S27:** ATAC-seq quantification used to separate patient tumors into expression groups and their *SOX2* expression levels.

**Supplementary Table S28:** Significantly correlated transcription factors to accessible chromatin at the SRR124–134 cluster in BRCA, LUAD, and LUSC tumors.

**Supplementary Table S29:** *FOXA1* transcript levels and chromatin accessibility at the SRR124–134 cluster in BRCA, LUAD, and LUSC patient tumors.

**Supplementary Table S30:** *NFIB* transcript levels and chromatin accessibility at the SRR124–134 cluster in BRCA, LUAD, and LUSC patient tumors.

**Supplementary Table S31:** Chromatin accessibility analysis in human (GRCh38/hg38) lung and stomach embryonic tissues.

**Supplementary Table S32:** Chromatin accessibility analysis in mouse (GRCm38/mm10) embryonic lung and stomach tissues.
